# Functional annotation of variants of the *BRCA2* gene via locally haploid human pluripotent stem cells

**DOI:** 10.1038/s41551-023-01065-7

**Published:** 2023-07-24

**Authors:** Hanqin Li, Rebecca Bartke, Lei Zhao, Yogendra Verma, Anna Horacek, Alma Rechav Ben-Natan, Gabriella R. Pangilinan, Netravathi Krishnappa, Rasmus Nielsen, Dirk Hockemeyer

**Affiliations:** 1 Department of Molecular and Cell Biology, University of California Berkeley, Berkeley, CA USA; 2 Innovative Genomics Institute, University of California, Berkeley, CA USA; 3 Section for GeoGenetics, Globe Institute, University of Copenhagen, Copenhagen, Denmark; 4 Chan Zuckerberg Biohub, San Francisco, CA USA

**Keywords:** Stem-cell biotechnology, Cancer genetics, CRISPR-Cas systems

## Abstract

Mutations in the *BRCA2* gene are associated with sporadic and familial cancer, cause genomic instability and sensitize cancer cells to inhibition by the poly(ADP-ribose) polymerase (PARP). Here we show that human pluripotent stem cells (hPSCs) with one copy of *BRCA2* deleted can be used to annotate variants of this gene and to test their sensitivities to PARP inhibition. By using Cas9 to edit the functional *BRCA2* allele in the locally haploid hPSCs and in fibroblasts differentiated from them, we characterized essential regions in the gene to identify permissive and loss-of-function mutations. We also used Cas9 to directly test the function of individual amino acids, including amino acids encoded by clinical *BRCA2* variants of uncertain significance, and identified alleles that are sensitive to PARP inhibitors used as a standard of care in *BRCA2*-deficient cancers. Locally haploid human pluripotent stem cells can facilitate detailed structure–function analyses of genes and the rapid functional evaluation of clinically observed mutations.

## Abstract (web summary)

Human pluripotent stem cells with one copy of the *BRCA2* gene deleted can be used to annotate variants of the gene and to test their sensitivities to inhibition by the poly(ADP-ribose) polymerase.

## Main

Genetic variants are key determinants of disease risk and can substantially impact diagnosis, prognosis and treatment outcomes. Classifying genetic variants of uncertain significance (VUS) in cancer genes such as *BRCA1* and *BRCA2* by PARP inhibitor sensitivity would result in clinically actionable, patient-specific information^1–3^. Recent advances in genome engineering have provided the necessary toolkit to introduce and evaluate disease-related variants at endogenous loci^4–11^. Using these tools, *BRCA* variants have previously been introduced into cancer cell lines using non-homologous end joining, homology-directed repair (HDR)^12^, or base editing^13^ or prime editing^14^ mediated mutagenesis.

A key limitation towards the implementation of these sophisticated genetic tools in the clinical interpretation of VUS is their use of cancer cell lines that inevitably harbour uncharacterized mutations in the DNA damage and repair pathways. These cell-line-specific idiosyncrasies alter cellular responses to perturbation and complicate interpretation and generalization of the results. A clinically relevant system would allow for the assessment of a *BRCA* variant in a genetically controlled and disease-relevant primary cell system through comparison of the phenotypic impact of a specific *BRCA* mutation with its isogenic counterpart without the mutation. As outlined by the American College of Medical Genetics and Genomics/Association for Molecular Pathology interpretation guidelines, clinical variants should be evaluated using information from several orthogonal approaches in genetically diverse backgrounds that reflect the pathophysiology of patient populations^15–17^. For example, in the case of familial forms of BRCA deficiency, such a system would evaluate allele-specific drug sensitivity of a cell that underwent loss of heterozygosity compared with a patient’s heterozygous non-cancer tissue^18,19^.

Several landmark studies^20–22^ have shown that human haploid cells can facilitate functional annotation of genes and genetic variants due to the strict genotype-phenotype relationship of single-allele alterations. Yet, haploid cells have a strong intrinsic tendency to spontaneously endoreduplicate and re-gain diploidy^20–22^, indicating genomic instability and an altered-physiological cellular state that compensates for an estimated 3,000 haploinsufficient genes in the human genome^23^. In this Article, we aim to develop a high-throughput, clinically actionable functional assay to investigate genetic variants in a genetically stable and physiology-representative human cell system.

## Results

**Fig. 1:**
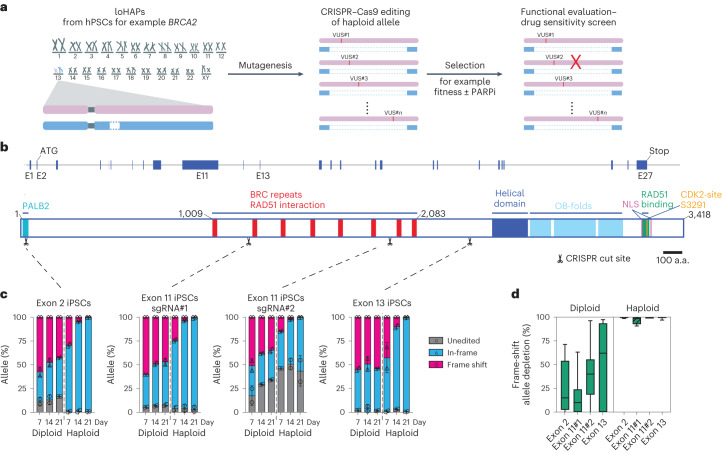
Annotating functional protein domains in *BRCA2* using CRISPR-mediated mutagenesis in *BRCA2* loHAPs. **a**, Annotation of genetic variants using loHAPs. First, one copy of a gene of interest (such as *BRCA2*) is excised from hPSCs to generate loHAPs. In a second step, the remaining allele is mutagenized (by introducing VUS) to identify alleles that are permissive and stably maintained in the cell pool. Finally, the pool of cells is challenged (for example, with a PARP inhibitor in *BRCA2* loHAPs) to identify variants sensitive to the treatment. **b**, Schematic representation of the *BRCA2* locus and the BRCA2 protein domain structure. Shown are the *BRCA2* exons (blue boxes), the sgRNA target sites used in this study (indicated with scissors) as well as the BRCA2 protein domain structure: PALB2 domain (turquoise), BRC repeats (red), helical domain (dark blue), OB-folds (light blue), C-terminal RAD51 binding domain (green), two nuclear localization signals (NLS, purple) and CDK2 phosphorylation sites (orange). Scale bar, 100 amino acids (a.a.). **c**, Quantification of categorized allele frequencies (unedited, in-frame and frame-shift indels) in exons 2, 11 and 13 assayed in diploid and *BRCA2* loHAP hiPSCs at days 7, 14 and 21 after editing. Bars showing mean of two biological replicates and error bars showing s.e.m. **d**, Quantification of the relative depletion of frame-shift mutation in exons 2, 11 and 13 comparing samples collected at day 7 and 21 after editing in diploid and *BRCA2* loHAP hiPSCs. Boxes showing quartiles and whiskers showing the 10th and 90th percentile. Mutations with frequency >0.5% are plotted. Source data

**Extended Data Fig. 1:**
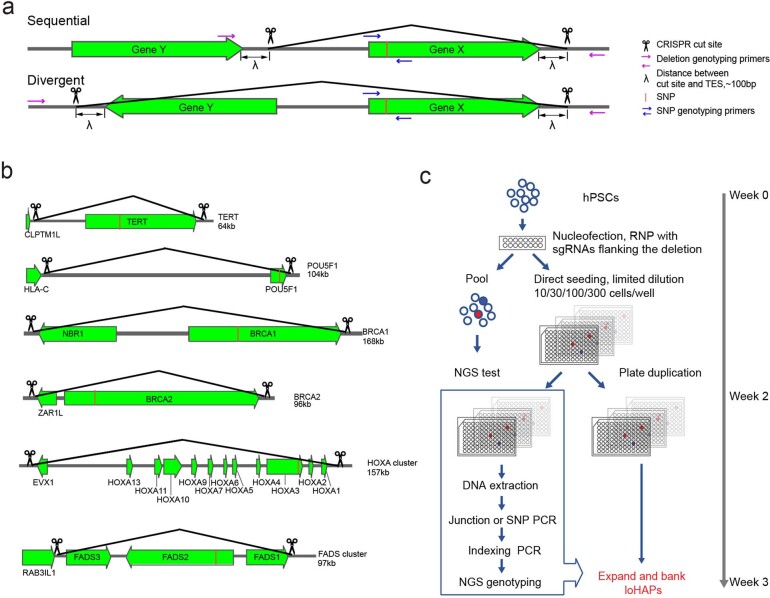
Design and gene-editing pipeline of loHAPs. **a**, General design of loHAPs, with key parameters. **b**, Specific design of loHAPs for each gene of interest, with deletion size specified. **c**, A scalable high-throughput gene editing pipeline combining limit dilution into 96-well right after nucleofection and multiplex NGS genotyping. Source data

Towards such assay, we took advantage of haploid genetics and developed a highly efficient method to generate locally haploid cells (loHAPs) from human embryonic stem cells (hESCs) and human-induced pluripotent stem cell (hiPSCs), collectively referred to as hPSCs. In loHAPs, one allele of the genomic region of interest is deleted, and genetic variants are introduced on the remaining functional allele, creating a haploid setting where mutations can be directly functionally tested (Fig. 1a). First, we designed an editing strategy that removes the entire gene of interest by excising the genomic regions between ~100 bp downstream of the transcriptional ends of the two neighbouring genes. Successful deletions are identified with polymerase chain reactions (PCRs) detecting the presence of de novo junctions and allele-specific single-nucleotide polymorphisms (SNPs) in the deleted region (Extended Data Fig. 1a,b). To isolate loHAPs, we used a gene editing pipeline that uses a limited dilution, and next-generation sequencing (NGS)-based genotyping strategy^24^ (Extended Data Fig. 1c) to generate clonal loHAPs for all six genes attempted with deletion sizes ranging between 64 kb and 168 kb. Our editing efficiencies ranged from 1% to 26.7%, assayed in one male iPSC cell and one female hESC line (*BRCA1*, 168 kb deletion, 2.9–24.9%; *BRCA2*, 96 kb deletion, 7.4–25.4%; *POU5F1*, 104 kb deletion, 19.9%; *HOXA* cluster, 157 kb deletion, 1%; *TERT*, 64 kb deletion, 26.7%; *FADS* cluster, 97 kb deletion, 8.6%) (Supplementary Fig. 1 and Supplementary Table 1). The notable high efficiency of loHAPs generation achieved non-virally using standard ribonucleoprotein (RNP) clustered regularly interspaced short palindromic repeats (CRISPR) delivery methods without the need for drug selection makes it a widely accessible and generalizable approach (for a detailed step-by-step protocol, see Methods). To demonstrate the power of loHAPs for functionally annotating VUS, we next focused on *BRCA2* loHAPs, which were generated in both hiPSCs and hESCs, proliferated normally and maintained pluripotency (Supplementary Fig. 2). LoHAPs remained haploid at the *BRCA2* locus without additional karyotypic difference compared with parental cells as assayed by array comparative genomic hybridization at passages 15–20.

The *BRCA2* gene comprises 27 exons and encodes a full-length BRCA2 protein of 3,418 amino acids with previously annotated protein domains of distinct functions^25,26^ (Fig. 1b). BRCA2 interacts at its N-terminus with PALB2 (amino acids 10–40, encoded by exons 2 and 3), contains eight BRCT repeats that can interact with RAD51, one α-helical domain and three oligonucleotide/oligosaccharide-binding folds (OB-folds) involved in single-stranded DNA binding^27^, and harbours a functionally distinct C-terminal RAD51 interaction domain (encoded by exon 27 and mapped to amino acids 3,280–3,305) (ref. ^25^). A frame-shift mutation in exon 27 that disrupted this interaction has been associated with patient cancer predisposition^28–31^. In contrast, truncating mutations at amino acid 3326 (K3326X) have been reported to be non-pathogenic^32,33^ or likely to have a low cancer risk^34^. Despite these insights, the exact truncation point at which BRCA2 function is substantially impaired, specifically which hypomorphic frame-shift mutations are sensitive to PARPi, remains largely unresolved^34^. About half of the 10,000 *BRCA2* variants listed in ClinVar^35^ are annotated as VUS, distributed over the entire length of the protein, confounding their functional and therapeutic interpretation.

**Extended Data Fig. 2:**
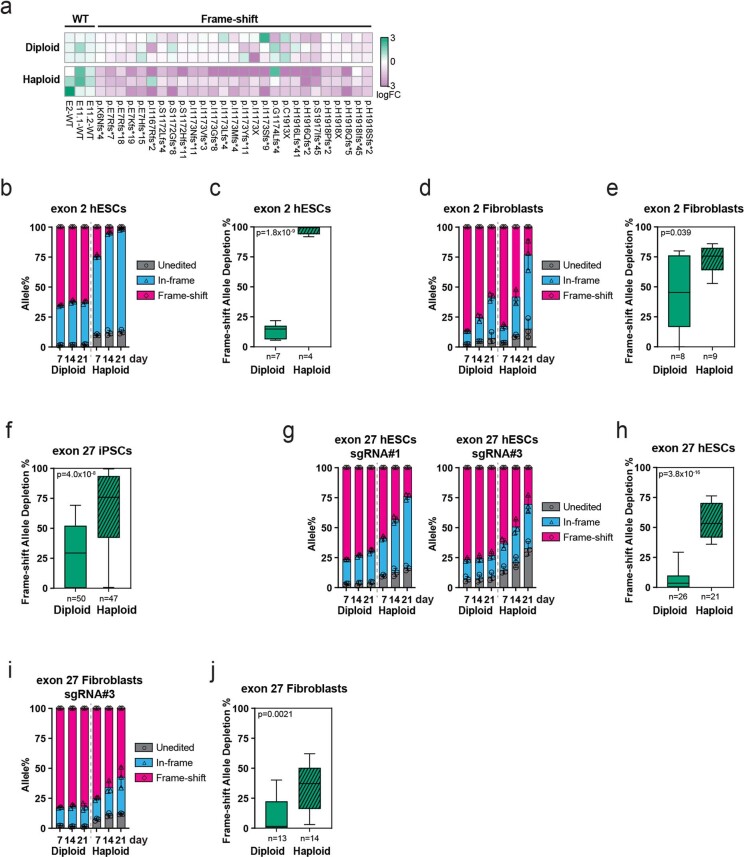
Annotating BRCA2 protein domain using CRISPR-mediated mutagenesis in loHAP hiPSCs, hESCs and hPSCs-derived fibroblasts. **a**, Heatmap indicating the allele frequency changes, log2 (day 14/day 7), of individual frame-shift mutation in 3 biological replicates in hiPSCs. **b**, Quantification of categorized allele frequencies (unedited, in-frame and frame-shift indels) for an sgRNA targeting exon 2 assayed in diploid and *BRCA2* loHAP hESCs at day 7, 14 and 21 after editing. Bars showing mean of 3 biological replicates and error bars showing SEM. **c**, Quantification of the relative depletion of frame-shift mutation in exon 2 comparing samples collected at day 7 and 21 after editing in diploid and *BRCA2* loHAP hESCs. Boxes showing quartiles and whiskers showing the 10th and 90th percentile. Mutations with frequency >0.5% are plotted. p, two-tailed Student’s t-test; n, number of alleles. **d**, Same as in **b**, for a sgRNA targeting exon 2 in hPSCs derived fibroblasts. **e**, Same as in **c**, for alleles generated in exon 2 in hPSCs derived fibroblasts. **f**, Same as in **c**, for alleles generated in exon 27 in hiPSCs. **g**, Same as in **b**, for sgRNAs targeting exon 27 in hESCs. **h**, Same as in **c**, for alleles generated in exon 27 in hESCs. **i**, Same as in **b**. for a sgRNA targeting exon 27 in hPSCs derived fibroblasts. **j**, Same as in **c**, for alleles generated in exon 27 in hPSCs derived fibroblasts. Source data

To assess the performance of our assay to test *BRCA2* genetic variants, we compared the efficacy with which we can annotate loss-of-function alleles in loHAPs compared with isogenic diploid cells. First, we introduced insertions and deletions (indels) via non-homologous end joining after CRISPR/Cas9-mediated cutting in the first coding exon, exon 2 and exons 11 and 13 with previously validated single guide RNAs (sgRNAs)^36^ in hiPSC-based loHAPs and diploid cells (Fig. 1c). All four sgRNAs tested revealed the same pattern in these experiments: in diploid cells, the unedited allele and in-frame indels increased during 3 weeks of culture to a total of about 50%, while frame-shift mutations slightly decreased. However, this overall enrichment of in-frame indels and unedited alleles was dramatically more pronounced in *BRCA2* loHAPs, resulting in an almost complete absence of frame-shift mutations after 3 weeks of culture (Fig. 1c,d and Supplementary Table 2). These results were highly reproducible when analysing specific alleles shared between diploid and *BRCA2* loHAPs (Extended Data Fig. 2a) and were confirmed when comparing hESC-based cell lines (Extended Data Fig. 2b,c). Loss of BRCA2 is associated with a very specific cancer spectrum^37,38^, probably because tissues differentially rely on homologous recombination (HR) to repair DNA breaks^39^. Thus, we tested if our system could functionally annotate *BRCA2* variants in cell types differentiated from hPSCs. We tested this by introducing indels in exon 2 of fibroblasts differentiated from diploid or *BRCA2* loHAPs (Extended Data Fig. 2d,e), which largely recapitulated the phenotypes seen in hPSCs. Specifically, the effect size of deleterious frame-shift mutations was significantly larger in loHAPs compared with diploid fibroblasts (Extended Data Fig. 2e). However, frame-shift mutations depleted slower in fibroblasts than in stem cells, probably reflecting fibroblasts’ slower proliferation rate. Together, these data demonstrate that the loHAPs system can rapidly identify loss-of-function variants in hPSCs and differentiated cell types.

**Fig. 2:**
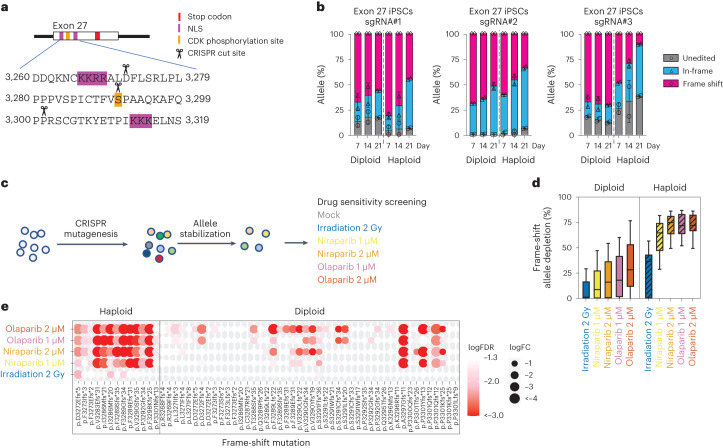
Annotating hypomorphic mutations in *BRCA2* exon 27 using CRISPR-mediated mutagenesis in *BRCA2* loHAPs. **a**, Overview of the domain structure of BRCA2 protein encoded by exon 27 (ref. ^25^). Indicated are two nuclear localization signals (NLS, purple) and a CDK phosphorylation site (orange) as well as three sgRNA cut sites used to mutagenize *BRCA2*. The BRCA2 stop codon is indicated in red. **b**, Quantification of categorized allele frequencies (unedited, in-frame and frame-shift indels) for sgRNAs targeting exon 27 assayed in diploid and *BRCA2* loHAP hiPSCs at days 7, 14 and 21 after editing. Bars showing mean of two biological replicates and error bars showing s.e.m. **c**, Schematic of the experimental design to identify hypomorphic *BRCA2* mutations. Cells (diploid or *BRCA2* loHAPs) are mutagenized using CRISPR-mediated editing. After allele stabilization (3–4 weeks after editing), cells are exposed to 2 Gy of gamma radiation, 1 µM or 2 µM of niraparib or olaparib or remain untreated (mock). Changes in allele frequency are quantified 1 week after treatment starts. **d**, Quantification of the relative depletion of frame-shift mutations in exon 27 after drug or irradiation treatment comparing diploid and *BRCA2* loHAPs. Boxes showing the quartiles. Whiskers defined as 10th and 90th percentile. Mutations with frequency >0.5% in at least one group are plotted. **e**, Drug responses of individual *BRCA2* exon 27 frame-shift mutations comparing *BRCA2* loHAPs and diploid hiPSCs. The size of each circle represents fold change (FC) of alleles in a given treatment against mock; colour intensity represents FDR-corrected one-tailed Student’s *t*-test *P* values. *n* = 6 in mock, *n* = 3 in the treatment groups; both were biological replicates. The same mutations as in **d** are plotted. Source data

A major clinical challenge is the classification of hypomorphic *BRCA2* variants that retain some BRCA2 function while still being sensitive to PARP inhibition. Thus, we next tested the hypothesis that loHAPs can be used to engineer and functionally annotate such hypomorphic alleles. To do this, we generated frame-shift mutations at three positions in exon 27 where hypomorphic alleles identified in genetically engineered mouse models^40,41^ and VUS are prevalent (Fig. 2a). We find that frame-shift mutations were more tolerated in exon 27 than in exon 2, and the enrichment for the unedited allele and in-frame indels was reduced. The unedited and in-frame indels increased to less than 50% in diploid cells after 3 weeks (Fig. 2b), and the increase of these functional alleles was again more pronounced in loHAPs than in diploid cells. Overall, frame-shift alleles in exon 27 had reduced fitness, yet a considerable proportion of frame-shift mutations were retained in *BRCA2* loHAP hiPSCs (Fig. 2b and Extended Data Fig. 2f), hESCs (Extended Data Fig. 2g,h) and differentiated fibroblasts (Extended Data Fig. 2i,j). To directly test the hypothesis that these remaining frame-shift alleles retained some functions yet are sensitive to PARP inhibition, we analysed the allele spectrum changes of loHAPs and diploid cells carrying frame-shift mutations in exon 27 when treated with 2 Gy of ionizing irradiation or two doses of the PARP inhibitors, niraparib and olaparib (Fig. 2c). Irradiation intensity and drug dosage were determined through dose–response curves on hPSCs (Supplementary Fig. 3a–c). Our analysis revealed that, while frame-shift mutations in exon 27 only mildly responded to irradiation, they were highly sensitive to treatment with niraparib or olaparib and depleted at all drug concentrations (Fig. 2d,e). Importantly, this depletion was much more pronounced in the *BRCA2* loHAPs than in the diploid cells (Fig. 2d). Further, analysis of individual frame-shift alleles showed that the penetrance of the effect was much more uniform in loHAPs than in diploid cells (Fig. 2e). This observation in the diploid cells is probably the consequence of a random expansion of compound heterozygotes carrying a frame-shift allele and a functional balancing allele that confound the effect of the mutation. These data suggest that *BRCA2* loHAPs can be used to reveal hypomorphic mutations that preserve residual BRCA2 function but are sensitive to PARP inhibition, providing an in vitro method to test clinical treatment options.

**Fig. 3:**
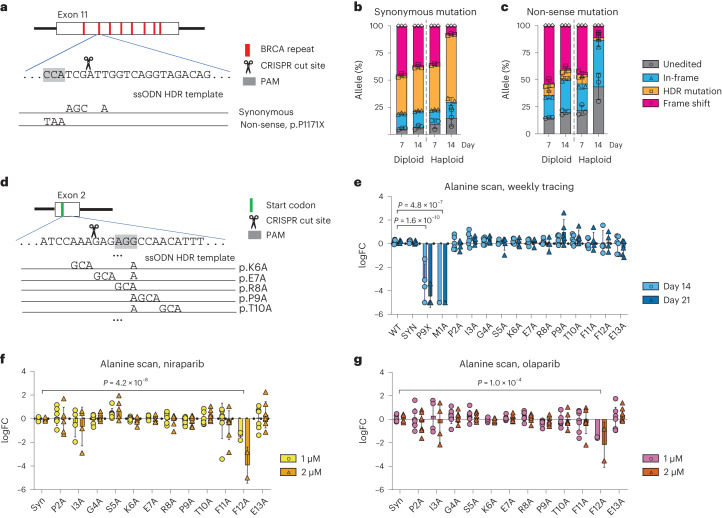
Using HDR-mediated genome engineering in *BRCA2* loHAPs to assess functionality of designer mutations. **a**, HDR-based editing strategy to introduce designer mutations in exon 11 of *BRCA2*. Shown is the targeted sequence with the PAM shaded in grey. Indicated below are the two HDR single-stranded repair oligos specifying the mutations that result in either synonymous or non-sense mutations. **b**, Quantification of categorized allele frequencies (unedited, in-frame indels, HDR-synonymous mutation and frame-shift mutations) in exon 11 assayed in diploid and *BRCA2* loHAP hiPSCs at days 7 and 14 after editing. Bars showing mean of three biological replicates and error bars showing s.e.m. **c**, Analysis as in **b**, using the HDR template introducing a non-sense mutation. **d**, HDR-based editing strategy to perform alanine scanning in *BRCA2*. Shown is the targeted sequence within exon 2 with the PAM shaded in grey. Indicated below are examples for the HDR single-stranded repair oligos, specifying the mutations that result in alanine replacements. The start codon is indicated in green. **e**, Allele frequency changes over time of mutations generated in *BRCA2* exon 2 alanine scanning in *BRCA2* loHAP hPSCs, represented as log_2_(day 14/day 7), circle, and log_2_(day 21/day 7), triangle. *P*, FDR-corrected two-tailed Student’s *t*-test versus WT. Bars showing mean and error bars showing s.e.m. Data points represent biological replicates. FC, fold change. **f**, Normalized allele frequency changes upon treatment of PARP inhibitor, niraparib, of *BRCA2* exon 2 alanine mutations in *BRCA2* loHAP hPSCs, represented as log_2_(drug/mock), 1 µM, circle; 2 µM, triangle. *P*, FDR-corrected two-tailed Student’s *t*-test versus synonymous mutations (Syn). Allele frequencies were normalized against corresponding WT. **g**, Same as in **f**, for olaparib treatment. Source data

**Extended Data Fig. 3:**
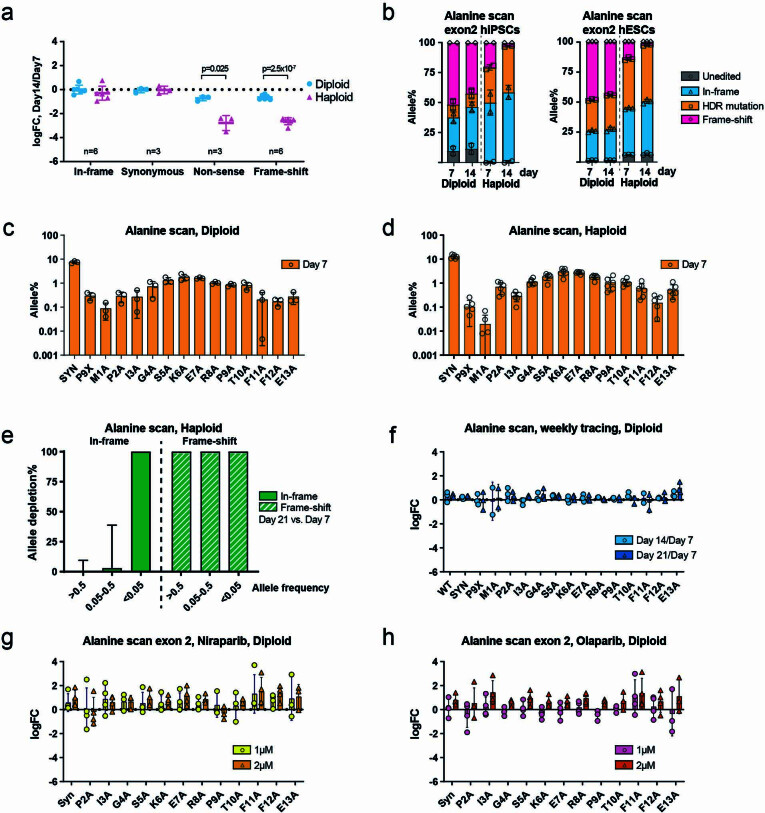
Alanine scanning in *BRCA2* loHAP hESCs and hiPSCs. **a**, Normalized allele frequency changes in categories (in-frame indels, synonymous, non-sense and frame-shift mutations) in exon 11 assayed in diploid and *BRCA2* loHAP hiPSCs, represent as log2 (day 21/day 7). P values report the significant comparisons between diploid and *BRCA2* loHAPs in each category, in two-tailed Student’s t-test. Centre showing mean and error bars showing SEM. n represents biological replicates. **b**, Quantification of categorized allele frequencies (unedited, in-frame indels, HDR alanine substitution and frame-shift mutations) in exon 2 assayed in diploid and *BRCA2* loHAP hiPSCs and hESCs at day 7 and 14 after editing. Bars showing mean of 2 or 3 biological replicates and error bars showing SEM. **c**, Allele frequencies of designer mutations in *BRCA2* exon 2 alanine scan in diploid hPSCs at day 7. Bars showing mean of 3 biological replicates and error bars showing SEM. **d**, Same as in **c**, for *BRCA2* loHAP hPSCs. **e**, Quantification of the relative depletion of in-frame and frame-shift mutations in different frequency groups in *BRCA2* loHAPs. Cells were edited in exon 2 performing alanine scanning as described in Fig. 3d–g and samples collected at day 7 and 21 after editing in *BRCA2* loHAP hPSCs. Frame-shift and in-frame alleles were binned into three groups based on their allele frequency (>0.5%, 0.05–0.5% and <0.05%) and the bars showing median and whiskers showing interquartile range. **f**, Allele frequency changes overtime of mutations generated in *BRCA2* exon 2 alanine scan in diploid hPSCs, represented as log2(day 14/day 7), circle and log2 (day 21/day 7), triangle. Bars showing mean and error bars showing SEM. Data points represent biological replicates. **g**, Normalized allele frequency changes upon treatment of PARP inhibitor, Niraparib, of *BRCA2* exon 2 alanine mutations in diploid hPSCs, represented as log2(drug/mock). 1 µM, circle; 2 µM, triangle. Allele frequencies were normalized against corresponding WT. Bars showing mean and error bars showing SEM. Data points represent biological replicates. **h**, Same as in **g**, for Olaparib treatment. Source data

A primary aim of our effort is to design a rapid workflow that gives clinicians an accurate method to identify VUS affecting BRCA function and test the efficacy of treatment with PARP inhibition. Thus, we established a methodology to assay the effect of variants by introducing them through HDR-mediated CRISPR editing. We first focused on *BRCA2* exon 11, this time providing single-stranded oligonucleotides (ssODN) as an HDR template that either introduced a synonymous or non-sense mutation (Fig. 3a). HDR-mediated introduction of a synonymous mutation was highly efficient for both diploid and *BRCA2* loHAPs. Notably, the abundance of non-sense mutation was considerably lower than the synonymous mutation at day 7, suggesting that some level of selection already occurred during the first week after editing (Fig. 3b,c). Regardless of this, the relative allele frequency of the synonymous mutation increased substantially more in *BRCA2* loHAPs than in diploid cells over time (Fig. 3b), consistent with our results for a functional allele in Fig. 1. By contrast, the mutant allele frequency of the HDR-introduced non-sense mutation dramatically decreased over time, an effect that was again significantly more pronounced in *BRCA2* loHAPs than diploid cells (Fig. 3c and Extended Data Fig. 3a).

To test the robustness of HDR-mediated mutagenesis, we introduced single amino acid substitutions in BRCA2 by performing alanine scanning experiments for the first 13 amino acids encoded by exon 2 and a non-sense substitution P9X as a complete loss-of-function control, using an ssODN pool encoding each alanine substitutions. The ssODN pools were designed to introduce synonymous PAM abrogating substitutions to prevent CRISPR recutting of alleles repaired by HDR (Fig. 3d). In our hands, HDR-based methods were highly efficient in both hESC and hiPSC (Extended Data Fig. 3b). All designer substitutions were introduced in diploid and *BRCA2* loHAPs (Extended Data Fig. 3c,d), with a frequency consistent with previous reports that demonstrate that editing efficiency decreased as the distance between designer substitutions and the cut site increased^42^. Like non-sense mutations in exon 11, the abundance of non-sense substitution P9X was much lower compared with the substitution P9A at day 7, suggesting that some level selection already occurs within the first week (Extended Data Fig. 3c,d). By binning in-frame and frame-shift mutations, alleles with frequency >0.05% can be reliably interpreted in these experiments (Extended Data Fig. 3e, and see Methods for detailed coverage calculations). Except for the start codon destroying M1A mutation, the relative allele frequencies were maintained for all alanine mutations over the 3 weeks, suggesting they are functional and do not cause a strong competitive disadvantage compared with the wild-type allele (WT) (Fig. 3e). However, when cultured in the presence of PARPi, cells with the F12A mutation were sensitive to both drugs, niraparib and olaparib (Fig. 3f,g). These findings are consistent with previous work that demonstrated amino acids within position 10–12 are indispensable for BRCA2’s ability to interact with PALB2, as only BRCA2 polypeptide spanning amino acids 10–250, but not a polypeptide of 13–250 can immunoprecipitate PALB2 (ref. ^43^). Of note, when we performed the same alanine scanning experiment in diploid cells, it failed to reveal the deleterious effect of P9X and M1A during the 3 week window (Extended Data Fig. 3f) and PARPi sensitivity of F12A (Extended Data Fig. 3g,h).

**Fig. 4:**
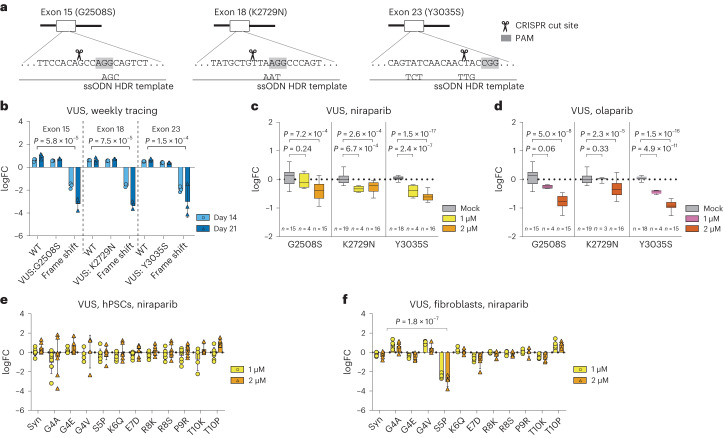
Using HDR-mediated genome engineering in *BRCA2* loHAPs to evaluate *BRCA2* VUS. **a**, HDR-based editing strategy to generate G2508S, K2729N and Y3035S. Shown are the targeted sequences with the PAM shaded in grey. Indicated below are the HDR single-stranded repair oligos used. **b**, Normalized allele frequency changes over time of G2508S, K2729N and Y3035S in *BRCA2* loHAP hPSCs, with WT and one frame-shift mutation at each locus as controls, represented as log_2_(day 14/day 7), circle, and log_2_(day 21/day 7), triangle. *P*, FDR-corrected two-tailed Student’s *t*-test versus WT. Bars showing mean of three biological replicates and error bars showing s.e.m. FC, fold change. **c**, Normalized allele frequency changes upon treatment of PARP inhibitor, niraparib, of G2508S, K2729N and Y3035S loHAP cell pools, represented as log_2_(drug/mock mean). *P*, FDR-corrected two-tailed Student’s *t*-test versus mock. Allele frequencies of G2508S and K2729N were normalized against corresponding WT; Y3035S were normalized against synonymous mutation. Boxes showing quartiles and whiskers showing the 10th and 90th percentile. *n* indicates biological replicates. **d**, Same as in **c**, for olaparib treatment. **e**, Normalized allele frequency changes upon treatment of PARP inhibitor, niraparib, of *BRCA2* exon 2 VUS in *BRCA2* loHAP hPSCs, represented as log_2_(drug/mock). 1 µM, circle; 2 µM, triangle. Allele frequencies were normalized against WT. Alleles with frequencies >0.5% in both hPSCs and fibroblasts are plotted. Bars showing mean and error bars showing s.e.m. Data points represent biological replicates. **f**, Same as **e**, in mutated *BRCA2* loHAP hPSC-derived fibroblasts. *P*, FDR-corrected two-tailed Student’s *t*-test versus synonymous mutations (Syn). Source data

**Extended Data Fig. 4:**
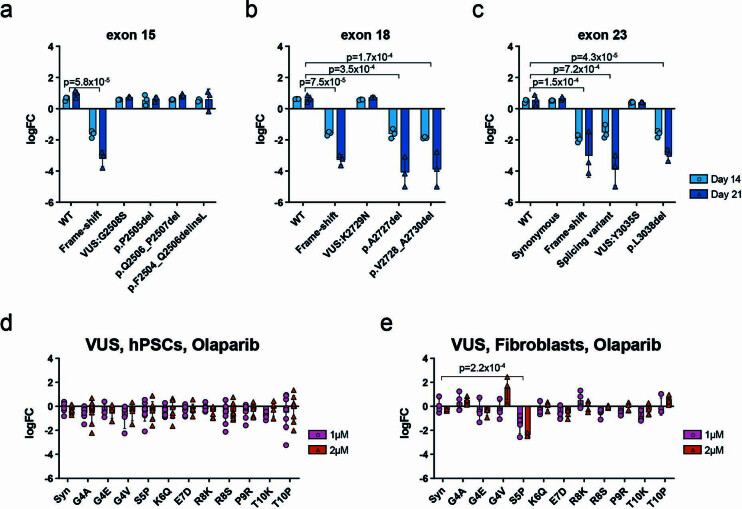
Allele frequency changes overtime of mutations generated around G2508, K2729 and Y3035 and drug sensitivity of VUS in hPSCs and fibroblasts. **a**, Allele frequency changes overtime of mutations generated around G2508 in *BRCA2* loHAP hPSCs. Represented as log2(day 14/day 7), circle and log2 (day 21/day 7), triangle. p, FDR-corrected two-tailed student’s t-test vs, WT. Bars showing mean of 3 biological replicates and error bars showing SEM. **b**, Same as **a**, for K2729. **c**, Same as **a**, for Y3035. **d**, Normalized allele frequency changes upon treatment of PARP inhibitor, Olaparib, of *BRCA2* exon 2 VUS in *BRCA2* loHAP hPSCs, represented as log2(drug/mock). 1 µM, circle; 2 µM, triangle. Allele frequencies were normalized against WT. Alleles with frequency >0.5% in both hPSCs and fibroblasts are plotted. Bars showing mean and error bars showing SEM. Data points represent biological replicates. **e**, Same as **d**, in mutated *BRCA2* loHAP hPSCs-derived fibroblasts. p, FDR-corrected two-tailed student’s t-test vs, synonymous mutations (Syn). Source data

Next, we benchmarked the sensitivity of our loHAP cells to reveal BRCA2 defects by testing three well-characterized hypomorphic mutations G2508S in exon 15, K2729N in exon 18, and Y3035S in exon 23 in loHAPs (Fig. 4a). These mutations have been shown to impose a moderate cancer risk that reflects their relatively mild defect in facilitating HR and in vivo mESC complementation assays^44^. Engineering these in-frame mutations into *BRCA2* loHAPs was highly efficient, so each mutation was introduced with a frequency of >24.5%. In contrast to frame-shift mutations, the frequency of all three mutations did not significantly depreciate over time compared with wild-type cells (Fig. 4b). Interestingly, in-frame deletions that were generated proximal to K2729 and Y3038 were depreciated, indicating that these regions of BRCA2 encode critical functions (Extended Data Fig. 4a–c). More importantly, when treated with niraparib and olaparib, all three mutations revealed a significant and dose-dependent sensitivity to PARP inhibition (Fig. 4c,d) that recapitulated the relative HR competence previously reported for these mutations^44^. Next, we evaluated cell-type-specific differences of VUS to PARP inhibition. We generated 11 VUS in *BRCA2* exon 2 in loHAP hPSCs using HDR-mediated mutagenesis and then differentiating the mutant hPSCs pool to fibroblasts. Exposing these cells to PARPi did not reveal any sensitivity in hPSCs (Fig. 4e and Extended Data Fig. 4d). However, when exposing the differentiated isogenic fibroblasts, we identified a cell-type-specific sensitivity of the S5P *BRCA2* mutations to niraparib and olaparib treatment (Fig. 4f and Extended Data Fig. 4e). Taken together, these results show that our assay has a sensitivity that is on par with the gold-standard assays for annotating *BRCA2* mutations^30,44,45^. Moreover, these data demonstrate that, through the differentiation of edited loHAPs, our assay can reveal cell-type-specific drug responses.

**Fig. 5:**
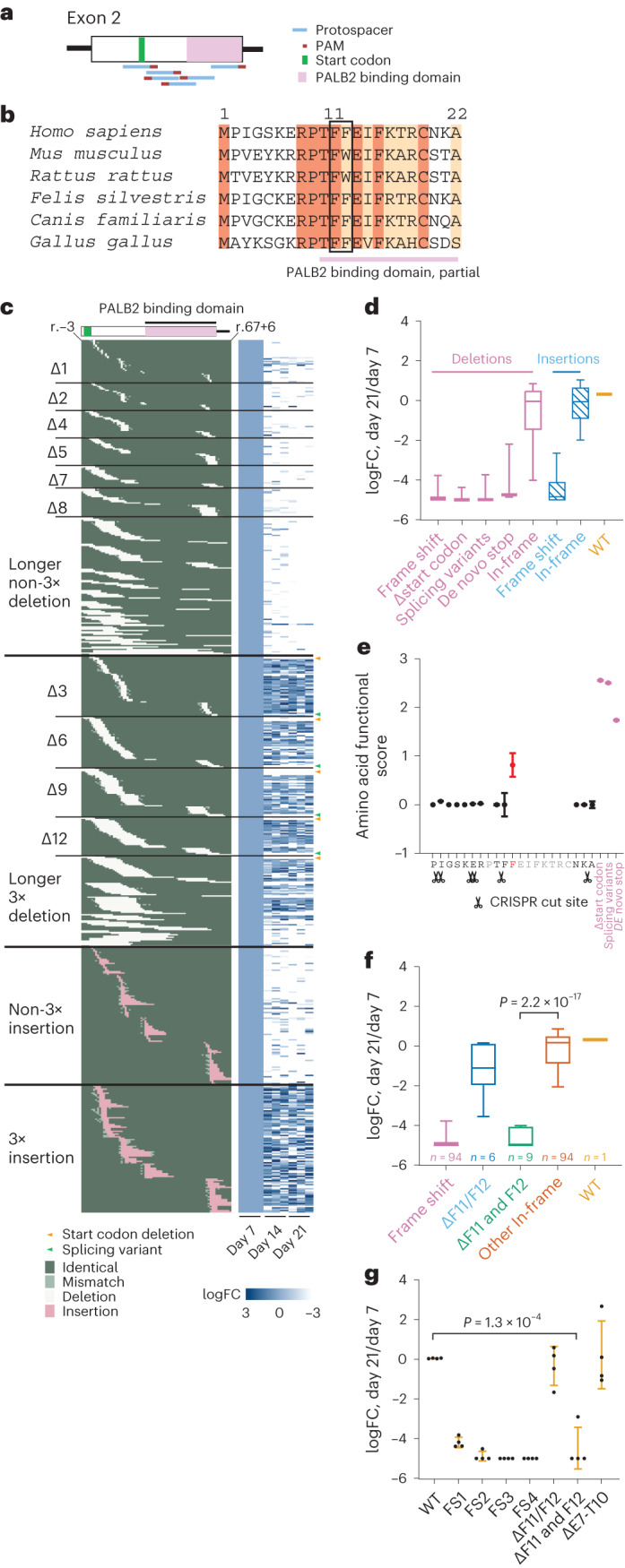
Using *BRCA2* loHAPs to identify critical amino acids by CRISPR-mediated mutation tiling in *BRCA2* exon 2. **a**, Schematic of editing strategy in the mutation tiling experiments on *BRCA2* exon 2. Shown are protospacers (blue), PAMs (red), start codon (green) and the part of PALB2 binding domain in exon 2 (pink). **b**, Sequence alignment of amino acids encoded by *BRCA2* exon 2 across multiple species. Identical amino acids are marked dark brown, and conserved amino acids are marked light brown. The pink bar indicates the part of PALB2 binding domain in exon 2. The black box highlights the identified critical amino acid F11, F12. **c**, The aligned indel profile and heatmaps of allele frequency change over time of each mutation at the nucleotide level in each of the triplicate experiments. Indel category is indicated to the left (3×, multiples of 3) and sample labels at the bottom. The identity of each position of each allele is categorized and colour coded as identical to WT (dark green), mismatch (light green), deletion (white) and insertion (pink). Specific mutations shown are start codon deletion (orange arrowheads) and splicing variant (green arrowheads). Allele frequencies of each mutation in each replicate were normalized to the corresponding day 7 frequencies. A total of 679 alleles that appeared in all independent day 7 samples and had at least 20 reads in at least one sample were analysed. FC, fold change. **d**, Categorized allele frequency changes of in-frame and frame-shift alleles in exon 2 at day 21 compared with day 7, presented as log_2_(day 21/day 7). Boxes showing quartiles and whiskers showing the 10th and 90th percentile. **e**, Functional score of each amino acid in *BRCA2* exon 2 calculated by a statistical model, with CRISPR cut site labelled. Only scores of amino acids covered by one amino acid deletion are plotted. The F12 with outstanding functional score is marked in red. Known detrimental mutation groups are shown in purple as positive controls. Dots indicate mean and error bars indicate standard deviation. **f**, Categorized allele frequency changes of F11, F12 deletions at day 21 compared with day 7, presented as log_2_(day 21/day 7). *P* value reports the significance between F11 and F12 double deletion (*n* = 9) versus other in-frame mutations (*n* = 94) in two-tailed Student’s *t*-test. Boxes showing quartiles and whiskers showing the 10th and 90th percentile. **g**, Normalized allele frequency changes of amino acid F11, F12 deletions at day 21 compared with day 7, presented as log_2_(day 21/day 7), in a targeted validation experiment, with allele category labels at the bottom. FS1-4, four examples for different frame-shift mutations and ΔE7-T10 for unaffected in-frame deletions. *P* value reports the significance between F11 and F12 double deletion versus WT in two-tailed Student’s *t*-test. *n* = 4 biological replicates. Error bar presents standard deviation. Source data

**Extended Data Fig. 5:**
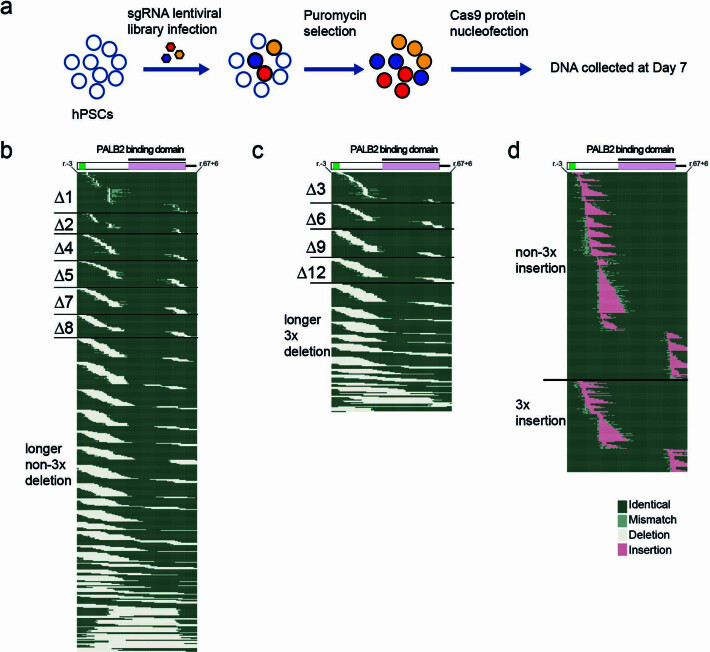
A representative indel profile of lentiviral sgRNA library-based mutation tiling in *BRCA2* exon 2. **a**, The workflow of lentivirus-based mutagenesis. **b**, The aligned deletion profile on nucleotide level of alleles with deletion size of non-multiples of 3 nucleotides, with category labels to the left. The identity of each position of each allele are categorized and color coded as identical to WT (dark green), mismatch (light green), deletion (white). **c**, Same as **b**, for deletions with deletion size of multiples of 3 nucleotides. **d**, Same as **b**, for insertions with inserted nucleotides in pink. Source data

**Extended Data Fig. 6:**
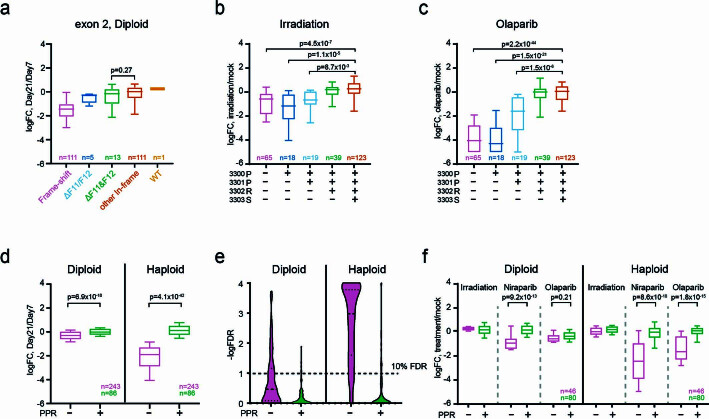
Comparison between diploid and *BRCA2* loHAPs in identifying functionally critical amino acids by CRISPR-mediated mutation tiling. **a**, Categorized allele frequency changes of F11, F12 deletions at day 21 comparing to day 7, presented as log2(day 21/day 7) in diploid. P value reports the significance between F11& F12 double deletion vs. other in-frame mutations in two-tailed Student’s t-test. n shown number of different alleles in each group. Boxes showing quartiles and Whiskers showing the 10th and 90th percentile. **b**, Categorized allele frequency changes of frame-shift mutations break before P3300, at P3300, at P3301, at R3302 or at later positions, upon irradiation comparing to the mock condition, presented as log2(irradiation/mock). Boxes showing quartiles and Whiskers showing the 10th and 90th percentile. P, FDR-corrected two-tailed student’s t-test vs. the PPRS fully preserved group. N, number of different alleles in each group. **c**, Same as **b**, for Olaparib treatment. **d**, Categorized allele frequency changes of frame-shift mutations with PPR motif preserved (+) or destroyed (−) assayed in diploid and *BRCA2* loHAP hESCs in an independent tiling deletion screen, at day 21 comparing to day 7, presented as log2(day 21/day 7). Boxes showing quartiles and Whiskers showing the 10th and 90th percentile. P, two-tailed student’s t-test. n, number of different alleles in each group. **e**, The distribution of FDR-corrected p value of each allele shown in **d**. The p values of alleles that frequencies increased, were assigned to be 1 (-logFDR=0). **f**, Same as **d**, upon treatment of irradiation, Niraparib or Olaparib comparing to the mock condition, presented as log2(treatment/mock). Source data

Thus far, our data show that loHAPs provide a highly sensitive genetic background for the functional annotation of *BRCA2*. Next, we evaluated the scale and resolution that loHAPs can be employed to annotate BRCA2 function by CRISPR-mediated mutation tiling^46^. To this end, we mutagenized *BRCA2* using every possible sgRNA (as determined by the spCas9 NGG protospacer adjacent motif) in exon 2 (Fig. 5a,b). We tested two approaches: (1) delivery of the editing components as RNPs and (2) infection of cells with a lentiviral library expressing that sgRNA followed by Cas9-protein transfection (Extended Data Fig. 5). While both approaches were highly effective, we focused on the RNP approach as it resulted in overall higher editing efficiencies than the lentiviral approach (~34% compared with ~12%). As before, we followed changes in the mutant allele spectra of three independent triplicates over 3 weeks. Deep sequencing, filtering and alignment of the resulting alleles revealed a highly complex mutation spectrum, including an almost complete tiling of in-frame deletions that generate single, two or three amino acid deletions (Δ3, Δ6, Δ9 nucleotides) as well as tiling of frame-shift mutations at almost each amino acid position close to CRISPR cut sites (Fig. 5c). Our analysis of exon 2 identified several classes of mutations that are strongly selected against: (1) frame-shift mutations, (2) in-frame deletions that delete the ATG encoding the translational start site, (3) deletions that alter the core splice-donor site at the end of exon 2, or (4) deletions that are within the *BRCA2* reading frame (Δ3, Δ6, Δ9 nucleotides, and so on) that generate de novo stop codons at the deletion site (Fig. 5d). Given this validation, we next generated a statistical model to analyse the high complexity of in-frame deletions to evaluate the functional contribution of individual amino acids in exon 2 of *BRCA2* (Fig. 5e). In brief, the method uses maximum likelihood to estimate the functional effects of mutations in each site, combining information from mutations covering multiple sites and taking amino acid similarity into account. A full description of the model is given in Supplementary Methods. In agreement with our previous result, this model also identified the phenylalanine at position 12 as a critical residue in BRCA2. Simultaneous deletion of both F11 and F12 showed a significantly greater impact than single amino acid deletion in both the initial mutation tiling experiments (Fig. 5f) and in a targeted follow-up validation experiment (Fig. 5g). Thus, we demonstrate through alanine substitution and in-frame deletions that amino acid F12 is important for BRCA2 function, probably by mediation of interactions with PALB2 (ref. ^43^). Noteworthily, the same tiling deletion experiment, when performed in diploid cells, failed to reveal the deleterious effect of the F11 and F12 double deletion (Extended Data Fig. 6a). From these experiments, we conclude that loHAPs can be used to map critical protein–protein interactions with single amino acid resolution.

**Fig. 6:**
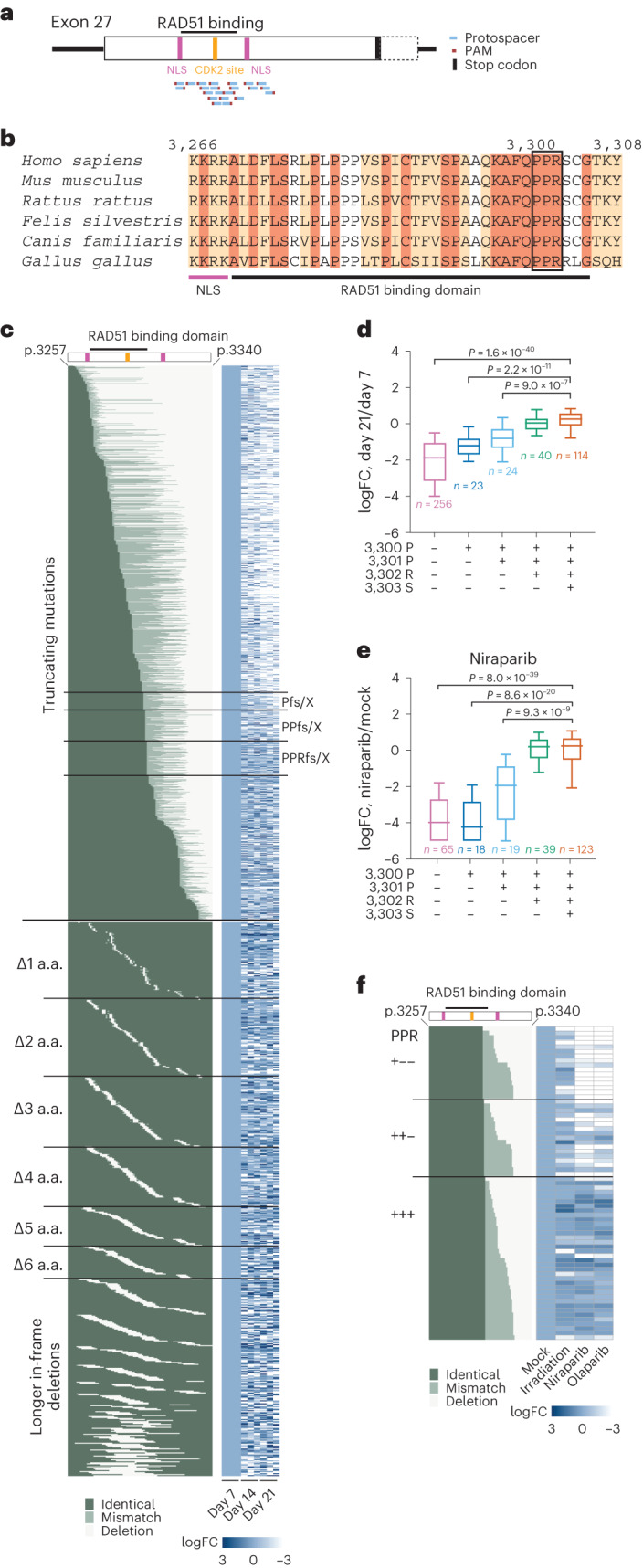
Using *BRCA2* loHAPs to identify critical amino acids by CRISPR-mediated mutation tiling in *BRCA2* exon 27. **a**, Schematic of editing strategy in the mutation tiling experiments on *BRCA2* exon 27. Shown are protospacers (blue), PAMs (red), stop codon (black), NLS (purple), CDK2 phosphorylation site (orange) and RAD51 binding domain (black bar on top). **b**, Sequence alignment of amino acids around the RAD51 binding domain (black bar) in exon 27 across multiple species. Identical amino acids are marked dark brown, and conserved amino acids are marked light brown. The first BRCA2 NLS is indicated with a purple bar. The black box highlights the identified critical amino acids PPR (3,300–3,302). **c**, The aligned full truncating mutation and in-frame deletion profile generated at amino acid (a.a.) level and heatmaps of allele frequency changes over time of each mutation, with mutation category labels to the left, sample labels at the bottom and the labels of truncating mutations (fs/X, frame-shift or de novo stop codon) break at the critical P3300, P3301 and R3302 at the right. The identity of each position of each allele is categorized and colour coded as identical to WT (dark green), mismatch (light green) and deletion (white). Allele frequencies of each mutation in each replicate were normalized to the corresponding day 7 allele frequencies. A total of 2,195 alleles that appeared in all independent day 7 samples and had at least 20 reads in at least one sample were analysed. **d**, Categorized allele frequency changes of frame-shift mutations before P3300, at P3300, at P3301, at R3302 or at later positions, at day 21 compared with day 7, presented as log_2_(day 21/day 7). Boxes showing quartiles and whiskers showing the 10th and 90th percentile. *P* values report the significant comparisons versus the last group in two-tailed Student’s *t*-test with FDR correction. *n*, number of different alleles in each group. Alleles with at least 100 reads at day 7 were analysed. FC, fold change. **e**, Categorized allele frequency changes on groups as in **d** upon niraparib treatment compared with the mock condition, presented as log_2_(niraparib/mock). Plotting parameters and statistics are shown the same as in **d**. Alleles with at least 100 reads in mock condition were analysed. **f**, The aligned allele profile at amino acid (a.a.) level of truncating mutations at P3300, P3301 or R3302, and heatmaps of allele frequency changes upon the treatment of irradiation or PARPi. Allele frequencies of each mutation were normalized to the corresponding allele frequencies in mock. Alleles with at least 100 reads in mock condition were analysed. Source data

Next, we tested if the mutation tiling approach can be used to map the specific amino acid position in exon 27 at which C-terminal truncating mutations render cells sensitive to PARPi. Mutagenizing loHAPs with 22 sgRNA cutting in exon 27 around the RAD51 binding domain (Fig. 6a,b) resulted again in a highly complex allele spectrum, with truncating and in-frame mutations tiling almost the entire region (Fig. 6c). While mutations at every position in exon 27 persisted until week 3 to some extent, only frame-shift mutations before R3302 showed a significant reduction in fitness (Fig. 6d). Subsequent exposure of these cells to 2 Gy irradiation only changed the allele spectrum mildly (Extended Data Fig. 6b). In contrast, treatment with both niraparib and olaparib independently revealed that frame-shift mutations before and at amino acid position 3302 are sensitive to PARPi. (Fig. 6e,f and Extended Data Fig. 6c). These results were validated in an independent deletion tilting screen (Extended Data Fig. 6d–f) in loHAPs and diploid cells. In diploid cells, the effects of frame-shift mutations binned into groups with and without the amino acid 3,300–3,302 PPR motif were much smaller compared with loHAPs (Extended Data Fig. 6d). Analysing individual mutations in loHAPs identified 86.0% of frame-shift mutations that truncate the amino acids 3,300–3,302 PPR motif as less competitive compared with wild type, while in diploid cells, only 30.0% showed a significant deleterious effect when correcting for a 10% false discovery rate (FDR) (Extended Data Fig. 6e). Similarly, the response to PARPi inhibition of variants without 3,300–3,302 PPR motif was inconsistent between niraparib and olaparib treatment, while both drugs had a significant effect in loHAPs (Extended Data Fig. 6f). Thus, predicting the performance of individual mutations is more reliable in loHAPs than in diploid cells.

**Extended Data Fig. 7:**
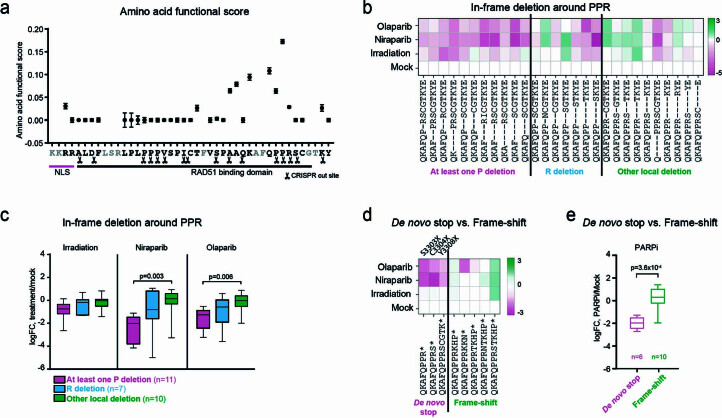
Evaluating the function of critical amino acids by analyzing in-frame deletions generated in CRISPR-mediated mutation tiling. **a**, Functional score of amino acid in the BRCA2 RAD51 binding domain encoded in exon 27 calculated by a statistical model, with CRISPR cut site labeled. Only scores of amino acids covered by one amino acid deletions are plotted. **b**, The aligned allele profile of <=4 amino acids in-frame deletions around PPR (3300–3302) and heatmaps of allele frequency changes upon the treatment of irradiation or PARPi. Alleles were grouped by the amino acid in PPR motif deleted. Allele frequencies of each mutation were normalized to the corresponding allele frequencies in mock. **c**, Categorized allele frequency changes of alleles in **b**. Boxes showing quartiles and Whiskers showing the 10th and 90th percentile. p, FDR-corrected two-tailed student’s t-test. n, number of different alleles in each group. **d**, The aligned allele profile of *de novo* stops and frame-shift mutation that result in stop codons between S3303 and Y3308 and heatmaps of allele frequency changes upon the treatment of irradiation or PARPi as shown in **b**. Note: We identified three *de novo* stop mutations S3303X, C3304X and Y3308X. In each case these *de novo* stops depleted after PARPi suggesting that *de novo* stop codons between 3302 and 3308 have reduced fitness suggesting that the presence of amino acids (even it not generated by the BRCA2 frame) between amino acid 3302 and 3308 contributes to BRCA2 protein function. These findings are consistent with a pathogenic *de novo* stop codon identified at amino acid 3308. **e**, Categorized allele frequency changes of alleles in **d** upon PARP inhibition. p, two-tailed student’s t-test. n, number of measurements in each group. The responses of each allele to Niraparib or Olaparib were considered as separate measurements. Source data

Our conclusion that amino acids 3301 and 3302 are critical amino acids in BRCA2 is again highly consistent with biochemical data that mapped the amino acids 3,280 to 3,305 in BRCA2 to be required for the recruitment of RAD51 (ref. ^25^) and with mouse data that map this interaction to the very same conserved region^47^ (P3301 *Homo sapiens* corresponds to P3224 *Mus musculus* (Fig. 6b)). To further corroborate our conclusion, we deployed our statistical model to evaluate the effect of in-frame mutations within the RAD51 binding domain (Fig. 6b) on BRCA2 function. Using the aforementioned statistical method for quantifying the effect of mutations in individual sites confirmed the importance of amino acids 3,293–3,302 and revealed overlap between evolutionarily conserved amino acids in this region (Extended Data Fig. 7a). Importantly, in-frame mutations that removed the 3,300–3,302 PPR motif and de novo stop mutations, S3303X, C3304X and Y3308X also rendered cells sensitive to PARPi (Extended Data Fig. 7b–e), which is consistent with the clinVar annotation and previous reports^28–31^. Thus, our data correlate the disruption of this well-mapped interaction domain as the most C-terminal frame-shift mutations that result in PARPi sensitivity.

## Discussion

We have established an efficient method to generate loHAPs in which large genomic regions of interest are excised from a single allele in hPSCs. Because only one copy of a gene of interest is present in loHAPs, allele frequencies in a cell population directly report on the fitness of mutations in response to an environmental or genetic perturbation. The pooled approach we use to assess mutations has several advantages: it allows direct comparison of mutant allele frequencies to the unedited WT and simultaneous testing of highly complex mutant pools with drugs classifying mutations into those responsive or resistant to the treatment.

Using the *BRCA2* gene, we provide proof of concept for functional annotation of mutations introduced on the remaining gene copy in loHAPs. We find mutations that disrupt the reading frame, delete the translational start site, introduce a de novo stop codon or impair proper splicing are annotated effectively in loHAPs. Moreover, we establish protocols that evaluate mutations introduced by HDR templates to perform alanine scanning experiments and investigate clinical VUS. When implemented in loHAPs, these protocols allow for more efficient evaluation of mutations compared with diploid counterparts. Moreover, we demonstrate that our pipeline can effectively annotate hypomorphic alleles that are functional in unchallenged cells but are sensitive to treatment with PARPi. For each of these experiments, we demonstrate that loHAPs outperform their diploid counterpart.

We perform these experiments in hESCs and iPSCs as well as differentiated cells, extending the range from the traditional cancer cell lines in which similar structure–function analyses are typically performed towards more diverse cell types with diverse genetic backgrounds. By extending the physiological context and genetic background of VUS analysis, our method can reveal cell-type-specific therapeutic responses of genetic variants. Thus, using loHAPs and their differentiated cell types can elucidate fundamental questions about why mutations with specific driver mutations, such as BRAC2, are associated with specific cancer types^37^. Together, our loHAPs assay to evaluate VUS fulfils the suggestions by the American College of Medical Genetics and Genomics/Association for Molecular Pathology clinical variant interpretation guidelines, which states that VUS evaluation ideally employs multiple orthogonal approaches reflecting the physiology, disease context and patient population^15–17^.

Our benchmarking experiments show that evaluating mutations loHAPs is robust, scalable and sensitive. We generate and test 13 continuous alanine substitutions in exon 2 of *BRCA2*. Moreover, by engineering three clinically relevant and well-characterized hypomorphic mutations, we demonstrate that our approach has a sensitivity that is similar in detecting defects as established complementation or direct HR activity assays to evaluate VUS in *BRCA2* (refs. ^30,44,45^).

Beyond the clinical evaluation of VUS, our proof-of-concept experiments highlight how functional annotation of genes in loHAPs can advance biological insights. By comprehensively mutating a region of the gene using every possible PAM, we generate a highly complex mutation spectrum that comprises in-frame deletions that almost completely tile across the region of interest. Using a new statistical model that can combine evidence from multiple mutations, each potentially spanning several sites, we demonstrate that this mutation tiling allows for the resolution of protein function to the single amino acid level. Specifically, we map the amino acids in *BRCA2* exon 2 required for the documented interaction with PALB2, the interacting region in exon 27 of *BRCA2* with RAD51, and the most C-terminal frame-shift mutation that renders cells sensitive to PARP inhibition.

Functionally annotating variants in loHAPs is generally limited to genes that are not fully haploinsufficient. Moreover, we find that the deletion spectrum in our tiling experiments is currently restricted by the availability of SpCas9 PAM sites. To fully cover the entire coding region of a gene might require deploying orthogonal Cas approaches^48,49^ that can cut at non-NGG protospacer adjacent motifs.

## Outlook

Locally haploid human pluripotent stem cells provide an approach for the evaluation of VUS in *BRCA2* and to test their sensitivity to PARPi in a cell-type-specific manner. Scaling this approach to the more than 5,000 reported VUS in *BRCA2* could provide a unique dataset to guide strategies for therapeutic interventions (Supplementary Fig. 4). Moreover, we expect that mutation scanning in loHAPs will prove as a general strategy to elucidate how mutations differentially trigger cell-type-specific responses. Finally, future experiments with loHAPs could be extended to analyse phenotypes more complex than cell survival and drug resistance.

## Methods

### Pluripotent stem cell culture

Pluripotent stem cell research is approved under 2012-12-024 by the Stem Cell Research Oversight Committee at the University of California, Berkeley. Human pluripotent stem cells (WTC11 (ref. ^50^) hiPSCs or WIBR3 (ref. ^51^) hESCs, National Institutes of Health stem cell registry #0079) were cultured on 4.1 × 10^5^ cm^−2^ irradiated mouse embryonic fibroblasts (MEFs) in hPSC medium (Dulbecco’s modified Eagle medium/Nutrient Mixture F-12 (DMEM/F12), 20% KnockOut Serum Replacement, 1× Non-Essential Amino Acids (NEAA), 1 mM glutamine, 1× penicillin/streptomycin, 0.1 mM β-mercaptoethanol and 4 ng ml^−1^ heat-stable basic fibroblast growth factor). The culture medium was changed daily, and cells were passaged with 1 mg ml^−1^ collagenase IV every 5–7 days. The day before and after passage, the medium was supplemented with 10 µM Y27632 (CD0141, Chemdea) to increase cell survival.

### Cas9/sgRNA RNP assembly

Chemically modified sgRNAs were purchased from Synthego. Cas9 protein was bought from the QB3 MacroLab, UC Berkeley. To assemble RNP, 300 pmol sgRNA and 80 pmol Cas9 were mixed in nuclease-free water to a final volume 10 µl, then incubated at room temperature (RT) for 5–10 min before nucleofection.

### Nucleofection of hPSCs and human fibroblasts

hPSCs cultured on MEF were detached from feeder cells by treating with 1 mg ml^−1^ collagenase IV and 0.5 U ml^−1^ dispase for 25–30 min. Detached colonies were washed with DMEM/F12 once and then dissociated to single cells following incubation with 1× acutase (SCR005, MilliporeSigma) for 5–7 min at 37 °C. Dissociated cells were washed with DMEM/F12 followed by a wash with phosphate-buffered saline (PBS). Next, 0.5 million hPSCs were pelleted then resuspended in 20 µl Lonza P3 primary nucleofection reagent, mixed with pre-assembled Cas9/sgRNA RNP with or without 100 pmol ssODN HDR donor, and nucleofected using program CA137 on Lonza nucleofector 4D. For nucleofection of hPSCs-derived fibroblasts, cells were dissociated with 0.25% trypsin–EDTA and then nucleofected with the same protocol.

### Generation of loHAPs

To generate loHAPs, we optimized previously reported genome editing strategies introducing large deletions^52–55^. Guide RNAs with specificity scores^56^ >70 were selected from a region 50–200 bp downstream of the annotated transcriptional end site of the gene of interest and the neighbour gene using the CRISPR Targets track^57^ in UCSC genome browser (Supplementary Table 3). The distance between the CRISPR cut site and transcriptional end site was empirically determined by weighing three factors: (1) avoiding indels that would disrupt gene functions on the remaining allele, (2) potential off-target effect when using guide RNAs with lower specificity scores and (3) preserving upstream *cis*-regulatory elements enabling future functional annotation of non-coding variants. As described above, a total of 300 pmol sgRNAs targeting the intended deletion region were delivered to hPSCs as RNPs by nucleofection. After nucleofection, cells were directly seeded onto four MEF 96-well plates at seeding densities of 10, 30, 100 and 300 cells per well, to compensate for batch-to-batch cell survival variability. The medium was changed at days 4, 7, 10, 12 and 13, and 10 µM Y27632 was supplemented at day 13. At day 14, cells were washed with PBS once and then treated with 40 µl 0.25% trypsin–EDTA for 5 min at 37 °C, and then 60 µl foetal bovine serum (FBS)/hPSC medium (replacing 10% KnockOut Serum Replacement with 10% FBS in regular hPSC medium) supplemented with 10 µM Y27632 was added to each well to inactivate trypsin. Cells were then gently triturated, and 50 µl cell suspension was transferred to a 96-well PCR plate pre-loaded with 50 µl 2× lysis buffer (100 mM KCl, 4 mM MgCl_2_, 0.9% NP-40, 0.9% Tween-20 and 500 µg ml^−1^ proteinase K, in 20 mM Tris pH 8) for DNA extraction. The remaining 50 µl of cells was reseeded to a new MEF 96-well plate pre-loaded with 100 µl 10 µM Y27632 supplemented FBS/hPSC medium and cultured for another 7 days with hPSC medium changed daily. Meanwhile, the cell lysate in the 96-well plates was incubated at 50 °C overnight and then heated to 95 °C for 10 min to inactivate the proteinase K. Cell clones were genotyped by detecting PCR amplicons spanning the deletion junctions using primer pairs flanking the deletion region and usually 100 bp away from the CRISPR cut site to capture potentially extended deletion might be caused by local homology. Amplicons were resolved by agarose gel electrophoresis, and Sanger sequenced or sequenced by NGS. Additionally, an SNP located within the deletion region in the hPSC cell line was identified from whole genome sequencing data. Using primers containing NGS barcode attachment sites (GCTCTTCCGATCT), the SNP region was amplified from 2 µl cell lysis from each well with Titan DNA polymerase. Amplicons were then purified using an automated SPRI beads purification protocol at the UC Berkeley DNA Sequencing Facility, i5/i7 barcoded, pooled and sequenced on 150PE iSeq or 300PE MiSeq in the NGS core facility at the Innovative Genomics Institute. NGS data were analysed using the CRISPResso2 (ref. ^58^) software to identify wells containing only one allele at the SNP. Cells in those wells identified to contain loHAPs were then subcloned by low-density seeding, manual picking and re-genotyped to establish clonal loHAP cell lines.

### Pluripotency marker staining

For immunofluorescent staining, cells were fixed with 4% paraformaldehyde in PBS for 20 min, permeabilized with 0.1% Triton X-100 in PBS for 30 min at RT, blocked in 3% bovine serum albumin in PBS for 1 h at RT and then incubated with primary antibody (PCRP-POU5F1-1D2, MC-813-70 (SSEA-4), Developmental Studies Hybridoma Bank) overnight at 4 °C, and secondary antibody (Thermo Fisher A11001) for 2 h at RT. For alkaline phosphatase staining, cells were fixed with cold 4% paraformaldehyde in PBS for 10 min, equilibrated with pH 9.5 100 mM Tris buffer for 10 min at RT, then incubated with NBT/BCIP (SK-5400, Vector Laboratories) at RT for 2 h. Images were acquired on a Zeiss Axio Observer A1 inverted fluorescence microscope.

### Human fibroblast differentiation from hPSCs

hPSC colonies were collected with 1 mg ml^−1^ collagenase IV, washed three times with 5% foetal calf serum in DMEM by gravitational settling, then cultured in suspension to form embryonic bodies in KSR medium (hPSC medium without human fibroblastic growth factor) in ultralow-attachment six-well plates. On day 4, the medium was changed to fibroblast medium (15% FBS in DMEM, 1× NEAA, 1× penicillin/streptomycin and 1× glutamine). At day 7, embryonic bodies were collected and seeded onto 0.2% gelatin-coated 10 cm dishes in fibroblast medium and medium was changed weekly until fibroblast morphology was established. Human fibroblasts were then passaged with 0.25% trypsin–EDTA every week with 1:3 splitting ratio.

### CRISPR-mediated mutagenesis

To introduce indels, 300 pmol sgRNA and 80 pmol purified Cas9 protein were delivered as RNP into hPSCs or hPSC-derived fibroblasts by nucleofection as described above. Targeted designer mutations were introduced with a 150–200 nt ssODN HDR template centred at the CRISPR cleavage site. HDR templates were designed with synonymous CRISPR–Cas9-blocking mutations to increase integration efficiency; when synonymous PAM-ablating mutations disrupted annotated regulatory sequences (for example, splicing sites) or were not feasible, sgRNA-blocking mutations were introduced proximal to the cut site. HDR templates contained a ≥2 nucleotide change relative to the WT sequence to distinguish designer mutations from sequencing errors. When introducing multiple variants (for example, alanine scanning) within a 30–40 nt window around the sgRNA cut site, ssODN templates were purchased from Integrated DNA Technologies (IDT) and nucleofected in the same HDR pool. In either case, 100 pmol of ssODN ultramer or oligo pool (customized oPools, IDT) were co-delivered with pre-assembled Cas9-RNPs (Supplementary Table 3) into 1 million cells. Cells were seeded back to multiple MEF wells as duplicates or triplicates after nucleofection and passaged with 0.25% trypsin–EDTA once a week as indicated (usually at days 7, 14 and 21 post-nucleofection) while a portion of cells was collected for genomic DNA during each passage. This experimental setting yielded ~25× coverage for a variant with a frequency 0.05% with a 5% post-nucleofection survival rate. With 5% empirical HDR efficiency, it allowed the introduction of 25–50 designer mutations in one single nucleofection. To determine allele frequencies in each cell pool, an approximately 250 bp region encompassing the CRISPR targeting site was amplified from 50–100 ng genomic DNA or cell lysis equivalent to ~10,000–20,000 genomes with primers containing NGS barcode attachment sites using Titan DNA polymerase or PrimeStar GXL DNA polymerase (R050B Takara). Amplicons were purified, barcoded and NGS sequenced, including an unedited control as described above. Alleles were called and quantified by CRISPResso2. For designer mutations that only differ by one nucleotide from WT, the baseline frequency caused by sequencing errors was measured relative to the unedited control.

### Generation of lentiviral sgRNA library

Oligonucleotides to clone lentiviral sgRNA expression vectors targeting exons 2 and 27 were purchased as oligo pools (IDT) cloned into BstXI/Blp1 (R0113S and R0585, NEB) double digested pJR104 (a gift from Jonathan Weissman) using NEBuilder HiFi DNA Assembly kit (E2627, NEB). Library complexity and quality were confirmed using NGS (each sgRNA had a coverage >100×). In total, 10 µg sgRNA library plasmids were transfected into ~8 million HEK293T cells together with 7.5 µg psPAX2 (#12260, Addgene) and 2.5 µg pMD2.G (#12259, Addgene) using Lipofectamine 2000 (11668019, ThermoFisher). Lentivirus was collected daily, filtered through a 0.45 µm filter, and frozen at −80 °C.

### Lentivirus-based mutagenesis

Human PSCs were detached from MEF by collagenase IV and dissociated to single cells by accutase as described above. One million cells were infected in suspension for 6 h with sgRNA library lentivirus at a multiplicity of infection of 0.15. Cells were then seeded onto puromycin-resistant MEF, selected by 0.8 µg ml^−1^ puromycin 48 h post-infection for one day, and expanded. A total of 100 pmol purified Cas9 protein was then delivered into 0.5 million infected cells by nucleofection, and cells were collected on day 7.

### Dose–response curve

For irradiation, 5 × 10^4^ hPSCs were irradiated in suspension at 0, 0.2, 0.5, 1, 2 and 5 Gy on Precision X-RAD 320 irradiator, then seeded back on MEF and cultured for 1 week. For PARP inhibitors, 5 × 10^4^ hPSCs were seeded on MEF and treated with 0, 0.5, 1, 2 and 5 µM niraparib (HY-10619, MedChem Express) or Olaparib (O-9201, LC Laboratories) for a week. Relative cell numbers in each condition were quantified by confluency using phase contrast images in ImageJ.

### Drug sensitivity screening of cells with *BRCA2* mutations

CRISPR-mutagenized cell pools were cultured for 3–4 weeks after mutagenesis to allow for the stabilization of the alleles present in the cell pools. Cells were then collected with 0.25% trypsin–EDTA as single cells. In triplicates, ~5 × 10^4^ cells were either irradiated in suspension with the indicated dose of gamma irradiation and seeded back onto MEF or seeded and then exposed to PARP inhibitor (niraparib or olaparib) using the indicated concentrations. Untreated samples with the same number of cells were seeded in parallel as mock controls. Cells were cultured for 1 week before genomic DNA was isolated. The mutagenized genomic regions in *BRCA2* were then amplified by PCR, NGS sequenced and analysed as described above. For drug sensitivity screening in fibroblasts, cells derived from hPSCs on days 30–50 post-differentiation were treated with PARPi for 10 days.

### RNP-based CRISPR-mediated mutation tiling

Each sgRNA was individually delivered into 1 million hPSCs as RNP, as described above. After nucleofection, cells were combined into two pools by the position of sgRNA they received (6 sgRNAs in exon 2, 22 sgRNA in exon 27), then each cell pool was seeded onto three 15 cm MEF dishes as biological triplicates. At least 0.3 million cells were replated for expansion or collected as genomic DNA every 7 days for 3 weeks. Drug sensitivity screening was started on day 21 and performed as described above, except that at least 0.3 million cells were used in each replicate, and cells were collected on day 28. At least 2 µg phenol–chloroform extracted genomic DNA from each sample was used as a template in PCR amplification of the mutagenized region, then samples were passed through the NGS pipeline as described above and sequenced at depth >1 million reads per sample on PE250 NovaSeq or PE150 NextSeq2000. NGS reads were first processed using CRISPResso2 to trim off adapter sequences, aligned to a provided reference sequence, and filtered by quality. After this, a custom Python script was used for further analysis. First, observed DNA deletions were categorized by proximity to the target cut site to separate out sequencing artefacts from rare gene editing events. If any part of an observed mutation was within two base pairs of a predicted CRISPR/Cas9 cut site, the allele progressed further into the analysis. If multiple mutations around cut sites were observed, the allele was filtered out, as the experiments outlined above do not permit the interpretation of complex editing events. If there were multiple technical replicates, defined here as independent PCRs performed from the same sample of genomic DNA, those read counts are combined into one file. Next, alleles were categorized into five groups. Alleles with mutagenized start codons are classified as ‘start codon deletions’, and alleles mutagenized at any nucleotide within ±3 bp around annotated splicing junctions were classified as ‘splicing variants’. The remaining alleles were further classified by their indel sizes. Alleles with indel sizes that were not multiples of 3 were classified as ‘frame-shift’ and alleles with indel size that were multiple of 3 were classified as ‘in-frame’, unless a novel stop codon formed at the junctions or within insertions, in which case they were classified as ‘de novo stop’. Then, alleles were bioinformatically translated on the basis of where the native splice site junctions are. Read counts from alleles with identical translations were combined to simplify downstream analysis. After this, only alleles that appeared in all mock or week 1 samples and had at least 20 reads in at least one sample were used for downstream analysis. We next normalized allele read counts from each file to the same allele in its respective biological replicate in mock or week 1. The in-frame deletions data were used to estimate the fitness of individual amino acids using a statistical model (Supplementary Methods).

### Data analysis and statistics

Hypothesis tests and multiple comparison correction were done as indicated using R 4.0. Bar graphs and box plots were drawn in GraphPad Prism 9. Heatmaps were generated using Morpheus (Broad Institute) or ggplot2 in R 4.0. Error bars indicate the standard error of mean (s.e.m.) unless otherwise specified.

### Reporting summary

Further information on research design is available in the Nature Portfolio Reporting Summary linked to this article.

### Supplementary information

Supplementary Information Supplementary Figs. 1–4, Tables 1–3, methods and references.

Reporting Summary

Supplementary code Custom Python code used in the mutation tiling experiments.

Supplementary Table 2 Ten most frequent indels present after 3 weeks post mutagenesis in diploid and loHAPs, as shown in Fig. 1c.

Supplementary Table 3 Sequences of sgRNAs and oligonucleotides used in this study.

### Source data

Source Data for Figs. 1–6 and Extended Data Figs. 1–7 Source data.

## Data Availability

The main data supporting the results in this study are available within the paper and its Supplementary Information. The raw sequencing data of the mutation-tiling experiments are available in the Gene Expression Omnibus repository under accession number GSE233683. Source data are provided with this paper.

## Code availability

The custom Python code used in the mutation tiling experiments is provided as Supplementary Information.
