# Supplementary material for: Functional annotation of variants of the BRCA2 gene via locally haploid human pluripotent stem cells
Source: Nat Biomed Eng. 2023 Jul 24;8(2):165–76. doi: 10.1038/s41551-023-01065-7 (PMC10878975; doi:10.1038/s41551-023-01065-7)
Supplement: Supplementary file 1 — Supplementary Figs. 1–4, Tables 1–3, methods and references. [file 41551_2023_1065_MOESM1_ESM.pdf]

nature biomedical engineering

Article

https://doi.org/10.1038/s41551-023-01065-7

Functional annotation of variants of the *BRCA2* gene via locally haploid human pluripotent stem cells

In the format provided by the authors and unedited

Contents

Supplementary Fig. 1 l Generation and characterization of loHAPs
Supplementary Fig. 2 l Pluripotency of the BRCA2 loHAP hiPSCs and hESCs.
Supplementary Fig. 3 l Dose-response curve of irradiation and PARP inhibitors on hPSCs.
Supplementary Fig. 4 l Schematic outlining a proposed application for loHAPs.
Supplementary Table 1 l The efficiencies for the generation of loHAPs in hPSCs.
Supplementary Table 2 l Table listing the 10 most frequent indels present after 3-weeks post mutagenesis in diploid and loHAPs as shown in Fig.1c.
Supplementary Table 3 l Sequences of sgRNAs and oligonucleotides used in this study.
Supplementary methods l Statistical modeling of in-frame deletions
Supplementary references

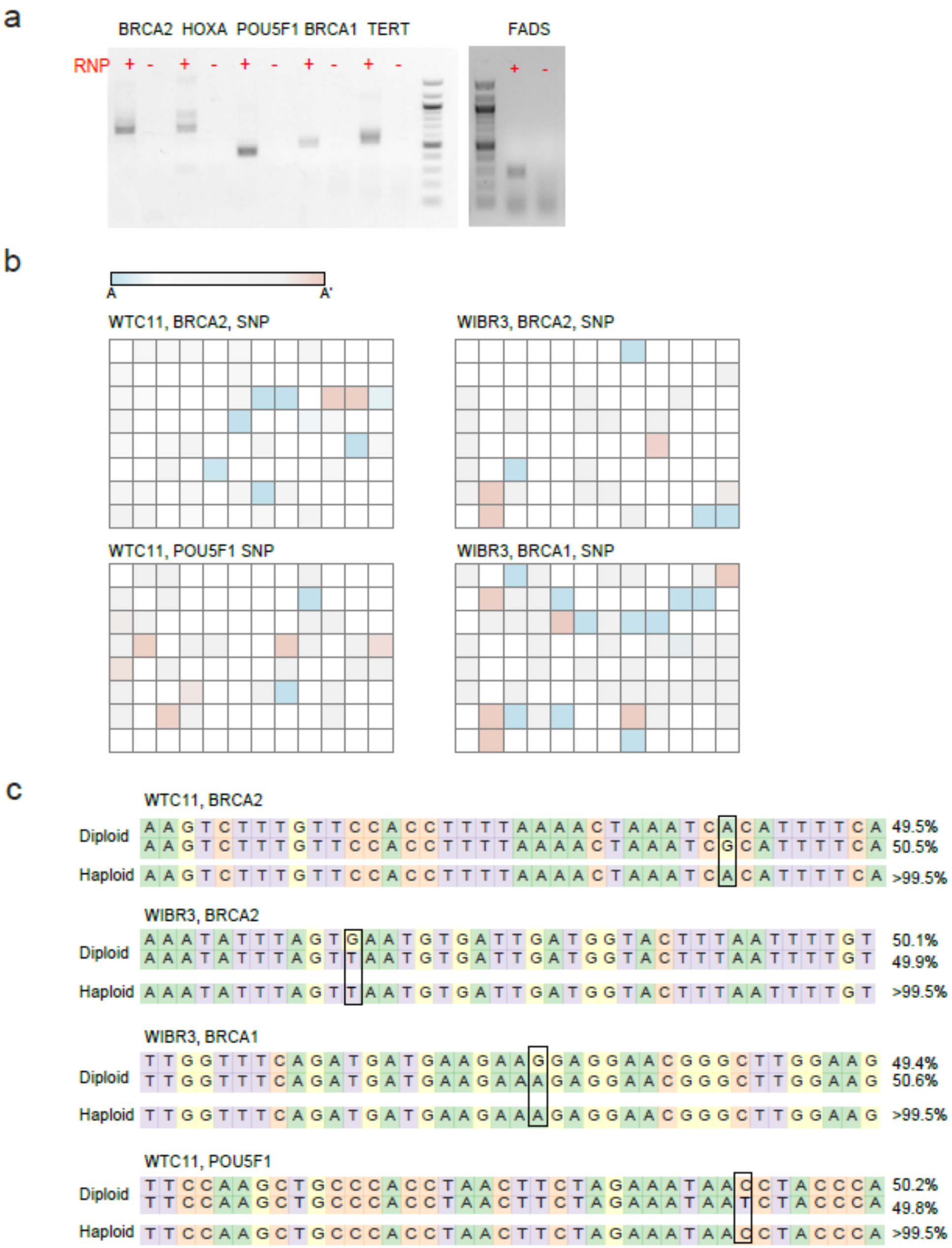

Supplementary Fig. 1 | Generation and characterization of loHAPs.

a, Detection of junctions of designed deletion for all 6 genes in bulk after CRISPR machinery delivered as RNP. **b,** Allelic profiles of the 96-well plates from which the loHAPs of BRCA1, BRCA2 and POU5F1 were isolated. Blue and red each represent a single allele (A and A' respectively). Wells which did not yield enough reads for accurate allele calling are uncolored. **c,** Allelic profiles of the final established loHAPs of BRCA1, BRCA2 and POU5F1 comparing to the parental diploid cells.

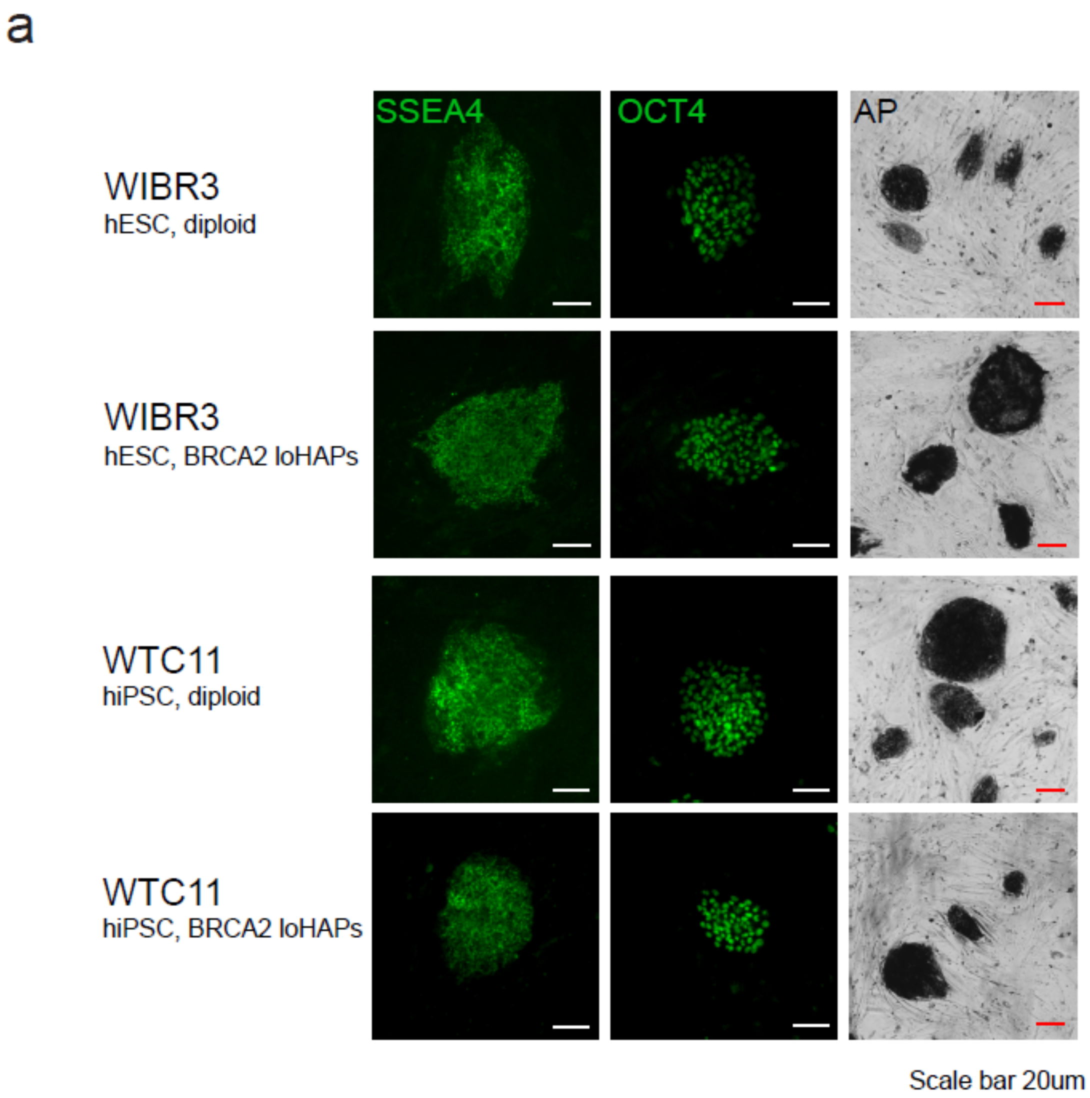

Supplementary Fig. 2 | Pluripotency of the BRCA2 loHAP hiPSCs and hESCs.
a, Immunostaining for pluripotency marker SSEA4 and OCT4 as well as histochemistry staining for the pluripotency marker alkaline phosphatase (AP). Scale bar, 20μm.

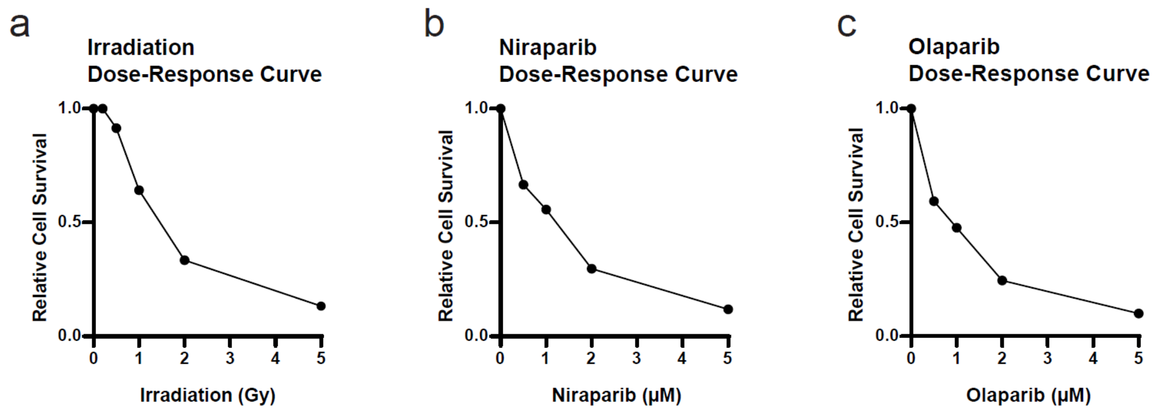

Supplementary Fig. 3 | Dose-response curve of irradiation and PARP inhibitors on hPSCs.
a, Dose-response of irradiation on hPSCs at 0, 0.2, 0.5, 1, 2 and 5 Gy. **b,** Dose-response of Niraparib on hPSCs at 0, 0.5, 1, 2 or 5μM. **c,** Dose-response of Olaparib on hPSCs at 0, 0.5, 1, 2 or 5μM.

a

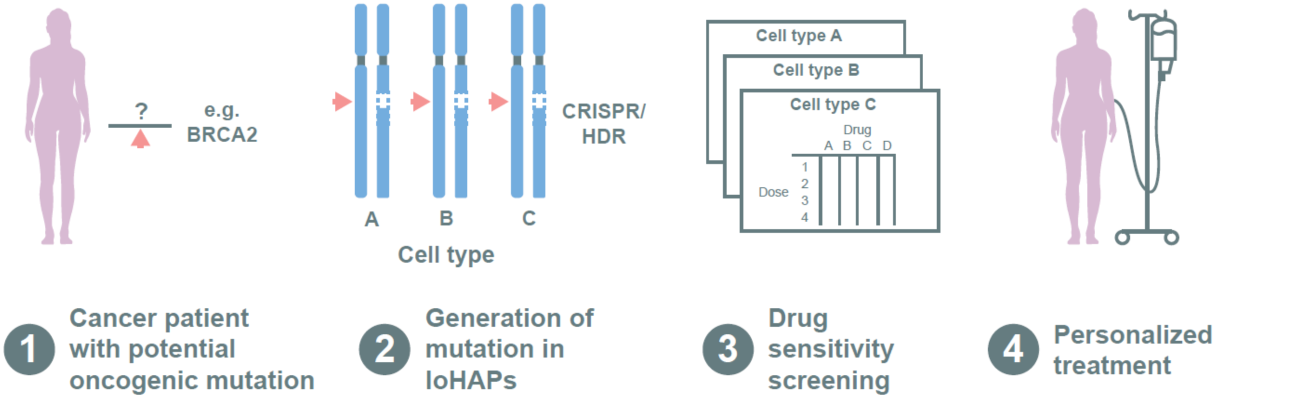

Supplementary Fig. 4 lSchematic outlining a proposed application for loHAPs. Rapid assessment of drug efficacy in multiple cell types in the context of patient specific mutations to guide therapeutic decision.

Supplementary Table 1 | The efficiencies for the generation of loHAPs in hPSCs.

Loci	Deletion length	Confirmed in WIBR3	Confirmed in WTC-11	loHAPs efficiency	Method measuring efficiency
BRCA1	168kb	x	x	2.9-24.9%	Deletion detecting PCR on manually picked clones or SNP PCR from 96-well plates
BRCA2	96kb	x	x	7.4-25.4%	Deletion detecting PCR on manually picked clones or SNP PCR from 96-well plates
POU5F1	104kb	x	x	19.9%	SNP PCR in 96-well plates
HOXA	157kb	n.a.	x	1%	Deletion detecting PCR in 96-well plates
TERT	64kb	x	x	26.7%	Deletion detecting PCR in 96-well plates
FADS	97kb	x	n.a.	8.6%	Deletion detecting PCR in 96-well plates

Supplementary Table 2 (separate file) | Table listing the 10 most frequent indels present after 3-weeks post mutagenesis in diploid and loHAPs as shown in Fig. 1c. Frame-shift mutations labeled in red.

Supplementary Table 3 (separate file) | Sequences of sgRNAs and oligonucleotides used in this study.

Supplementary methods | Statistical modeling of in-frame deletions

This section describes the method used for estimating the fitness effect of each site in a genomic region. We assume data of counts of amino acid haplotypes (including the null haplotype without mutations) across three time points (week 1, week 2, and week 3) in three independent experiments (replicate 1, replicate 2 and replicate 3). We model changes in haplotype frequencies as a function of their relative fitness while accounting for the randomness introduced by the sampling process.

All mutant haplotypes, without frame-shift indels, are first aligned against the reference and a functional distance between the reference base and the observed base (or deletion) is defined for all positions. For amino acid changing mutations, the distance is defined as Grantham's distance[1], rescaled so that the maximum value is 1. For deletions, the distance is defined as 2. If there is no mutation, the distance is 0. These distances define a matrix, D, with row i, d_i, representing haplotype i. We also define a fitness effect column vector, f, whose elements reflect the fitness contributions per distance per week of the corresponding site. d_i is haplotype specific, while f is assumed constant across all haplotypes, and is the parameter of interest that quantifies the relative importance of each site for the function of the protein. Assuming multiplicativity of fitness effects among sites, the expected frequencies of haplotype *i* at week 2 and week 3 of replicate *k* (*k*=1,2,3), denoted as $x_{1,i,k}$ and $x_{2,i,k}$, are

$$\begin{cases} x_{1,i,k} = \frac{y_{i,k}e^{-d_i f}}{\sum_j y_{j,k}e^{-d_j f}} \\ x_{2,i,k} = \frac{y_{i,k}e^{-cd_i f}}{\sum_j y_{j,k}e^{-cd_j f}} \end{cases} \quad (1)$$

where $y_{i,k}$ is the initial frequency (week 1) of haplotype *i* in replicate *k*, and *c* is the time scale factor between week 3 and week 2. We estimate $y_{j,k}$ directly from the data at week 1 and define a log-likelihood function for f and c, assuming multinomial sampling, as

$$l(f,c) = \sum_{i,k}\{n_{1,i,k}\log(x_{1,i,k}) + n_{2,i,k}\log(x_{2,i,k})\} \quad (2)$$

where $n_{1,i,k}$ and $n_{2,i,k}$ represent the counts of haplotype *i* at week 2 and week 3 of replicate *k*. Assuming that the introduced mutations have only negative selective effects (positive values of the elements of *f*), we optimize $l(f,c)$ with the constraints $f, c \geq 0$. To approximate the variance in the estimates, we use the Fisher information matrix at the optima of $l(f,c)$ in Equation 2).

Note that we can also estimate the fitness of a group of haplotypes based on this model. When estimating the fitness of a collection of haplotypes, we add one extra element to the vector d_i, which is an indicator of membership of the focal group. If haplotype *i* is within that group, we set all other elements of d_i to 0.

If multiple independent experiments for overlapping genomic region have been conducted, resulting in multiple datasets (e.g., $\mathfrak{D}_1$, $\mathfrak{D}_2$ and $\mathfrak{D}_3$ on exon 27), we optimize the following joint log-likelihood function for all *s* data sets:

$$l(f, c_1, \ldots, c_s) = \sum_{m=1}^{s} l_{\mathfrak{D}_m}(f, c_m). \quad (3)$$

subject to the condition $f, c_m \geq 0$.

Data processing

Different haplotypes with start codon deletion, *de novo* stop or splicing variant mutations were all grouped into one of these three categories, and we did not attempt to estimate fitness effects of different haplotypes within each category. Similarly, in cases where other mutations occurred on these haplotypes, these other mutations were not included in subsequent analyses of fitness effects. The implicit assumption is that mutations in the start codon deletion, *de novo* stop and splicing variant categories have such large fitness effects that the additive effect of subsequent mutations is negligible.

The alignments are done using Needleman-Wunsch algorithm[2] with scores given by the rescaled Grantham's distance[1]and gap-open and gap-extension penalties of 2 and 0.5, respectively. The reference for the alignment is the null allele. If only one optimal alignment is obtained, d_i is calculated as described above. If multiple alignments are equally optimal, d_i will be calculated based on the average distance between every optimal alignment and the null allele.

To further process the data, we first remove uninformative sites where all haplotypes have the same allelic state. We then collapse sites with mutation patterns that are not linearly independent. These sites are often neighboring sites that share the same deletion event(s), but other configurations might also lead to a lack of linear independence. To collapse sites, we implement an algorithm to identify a maximum set of sites that are linearly independent so that the matrix D will have full column rank. The estimates of *f* which correspond to the unremoved correlated sites, will then not represent the fitness effect of a single site, but rather a linear combination of the fitness effects of all the sites correlated with the focal one. The sets of collapsed sites are represented by a matrix $RelMat$. The updated version of D and the matrix $RelMat$, are found using the following algorithm:

**Input:** Matrix D
**Output:** Updated matrix D, and Relationship matrix $RelMat$
Obtain the total column number of D, N; Obtain the rank of D, R;<br>**Construction and Initialization:**<br>Construct a rank vector r and an index vector l, both of length N;<br>Construct a matrix $RelMat$ of size $R \times N$;<br>Initialize all elements of l to 1, and all elements of $RelMat$ to 0; $r[1] = 1$;<br>**Calculation:**<br>**For** $i = 2{:}N$<br>Calculate $r[i] = rank(D[,1{:}i])$;<br>**If** $r[i] == r[i-1]$<br>$l[i] = 0$;<br>$index = 1{:}(i-1)$;<br>Project $D[,i]$ on the linear space spanned by $D[,index[l[1{:}(i-1)]]]$,<br>and the corresponding coefficients form a column vector v;<br>$RelMat[1{:}sum(l[1{:}(i-1)]), i] = v$;<br>**Else**<br>$RelMat[sum(l[1{:}i]), i] = 1$;<br>**Endif**<br>**Endfor**<br>Update $D = D[,l > 0]$;<br>Return D and $RelMat$;

In this algorithm, $D[,1{:}i]$ denotes the matrix formed by the first i columns of D.

We then optimize the likelihood function in Equation (2) or (3) using the L-BFGS-B algorithm imbedded in the 'optim' function in R to obtain $\hat{c}$ and the fitness effect vector $\hat{f}$.

Supplementary references

1. Grantham, R. Amino acid difference formula to help explain protein evolution. *Science (New York, N.Y.)* **185**, 862-864 (1974).
2. Needleman, S.B. & Wunsch, C.D. A general method applicable to the search for similarities in the amino acid sequence of two proteins. *Journal of molecular biology* **48**, 443-453 (1970).
