# Supplementary material for: Functional annotation of variants of the BRCA2 gene via locally haploid human pluripotent stem cells
Source: Nat Biomed Eng. 2023 Jul 24;8(2):165–76. doi: 10.1038/s41551-023-01065-7 (PMC10878975; doi:10.1038/s41551-023-01065-7)
Supplement: Supplementary file 2 — Reporting Summary [file 41551_2023_1065_MOESM2_ESM.pdf]

nature portfolio

Corresponding author(s): Dirk Hockemeyer

Last updated by author(s): Jun 2, 2023

Reporting Summary

Nature Portfolio wishes to improve the reproducibility of the work that we publish. This form provides structure for consistency and transparency in reporting. For further information on Nature Portfolio policies, see our Editorial Policies and the Editorial Policy Checklist.

Statistics

For all statistical analyses, confirm that the following items are present in the figure legend, table legend, main text, or Methods section.

n/a	Confirmed	
☐	☒	The exact sample size (*n*) for each experimental group/condition, given as a discrete number and unit of measurement
☐	☒	A statement on whether measurements were taken from distinct samples or whether the same sample was measured repeatedly
☐	☒	The statistical test(s) used AND whether they are one- or two-sided<br>*Only common tests should be described solely by name; describe more complex techniques in the Methods section.*
☒	☐	A description of all covariates tested
☐	☒	A description of any assumptions or corrections, such as tests of normality and adjustment for multiple comparisons
☐	☒	A full description of the statistical parameters including central tendency (e.g. means) or other basic estimates (e.g. regression coefficient) AND variation (e.g. standard deviation) or associated estimates of uncertainty (e.g. confidence intervals)
☐	☒	For null hypothesis testing, the test statistic (e.g. *F*, *t*, *r*) with confidence intervals, effect sizes, degrees of freedom and *P* value noted<br>*Give P values as exact values whenever suitable.*
☒	☐	For Bayesian analysis, information on the choice of priors and Markov chain Monte Carlo settings
☒	☐	For hierarchical and complex designs, identification of the appropriate level for tests and full reporting of outcomes
☒	☐	Estimates of effect sizes (e.g. Cohen's *d*, Pearson's *r*), indicating how they were calculated

Our web collection on statistics for biologists contains articles on many of the points above.

Software and code

Policy information about availability of computer code

Data collection	Zen 2012 (blue edition), iSeq 100 Software System Suite v2.0, Illumina BaseSpace Sequence Hub
Data analysis	CRISPResso2 2.0.40, R 4.0.2, Graphpad Prism 9, Image J, Morpheus, ggplot2 3.3.5, python 3.7

For manuscripts utilizing custom algorithms or software that are central to the research but not yet described in published literature, software must be made available to editors and reviewers. We strongly encourage code deposition in a community repository (e.g. GitHub). See the Nature Portfolio guidelines for submitting code & software for further information.

Data

Policy information about availability of data

All manuscripts must include a data availability statement. This statement should provide the following information, where applicable:

- Accession codes, unique identifiers, or web links for publicly available datasets
- A description of any restrictions on data availability
- For clinical datasets or third party data, please ensure that the statement adheres to our policy

The main data supporting the results in this study are available within the paper and its Supplementary Information. Source data for the figures are provided with this paper. The raw sequencing data of the mutation-tiling experiments are available in the GEO repository under accession number GSE233683.

Research involving human participants, their data, or biological material

Policy information about studies with human participants or human data. See also policy information about sex, gender (identity/presentation), and sexual orientation and race, ethnicity and racism.

Reporting on sex and gender	The study did not involve human participants.
Reporting on race, ethnicity, or other socially relevant groupings	—
Population characteristics	—
Recruitment	—
Ethics oversight	—

Note that full information on the approval of the study protocol must also be provided in the manuscript.

Field-specific reporting

Please select the one below that is the best fit for your research. If you are not sure, read the appropriate sections before making your selection.

☒ Life sciences ☐ Behavioural & social sciences ☐ Ecological, evolutionary & environmental sciences

For a reference copy of the document with all sections, see nature.com/documents/nr-reporting-summary-flat.pdf

Life sciences study design

All studies must disclose on these points even when the disclosure is negative.

Sample size	We compared allele frequencies in n1 >= 6 null samples to n2 >= 3 samples under treatment. The same null samples were used for all treatments. The test employed was aimed at detecting differences in allele frequencies between null and treatment (h0: allele frequency in null = allele frequency under treatment). Because the sample sizes (number of reads) were relatively large, we used a t-test as an approximation. Assuming that reads were sampled according to a Poisson distribution, and that the number of reads was much smaller than the number of cells from which DNA was extracted, the variance in the number of reads with a mutation should equal the expected number of reads with that mutation. We used this observation for power calculations. For example, with expected 1,000 reads, the probability of detecting a systematic reduction from an allele frequency of 0.2 to 0.15 of a mutation using a one-sided t-test is 0.999 (calculated using the function pwr.t2n.test in R with Cohen's D calculated using the expected Poisson variance). The sample sizes used clearly provide high power to detect allele-frequency differences. In fact, the main reason for this level of replication is for reproducibility and to be able to accommodate increased variance caused by unknown confounders.
Data exclusions	Samples that did not yield PCR amplification and NGS data were excluded.
Replication	Measurements were taken from replicates. Key results were replicated with different sgRNAs and in different cell lines.
Randomization	Cells were equally split into sub-populations of 50,000 cells, which is at 250x coverage of an allele at 0.5% frequency. Those subpopulations were randomly assigned to different treatment groups.
Blinding	The experiments were open-labelled.

Reporting for specific materials, systems and methods

We require information from authors about some types of materials, experimental systems and methods used in many studies. Here, indicate whether each material, system or method listed is relevant to your study. If you are not sure if a list item applies to your research, read the appropriate section before selecting a response.

Materials & experimental systems

n/a	Involved in the study	
☐	☒	Antibodies
☐	☒	Eukaryotic cell lines
☒	☐	Palaeontology and archaeology
☒	☐	Animals and other organisms
☒	☐	Clinical data
☒	☐	Dual use research of concern
☒	☐	Plants

Methods

n/a	Involved in the study	
☒	☐	ChIP-seq
☒	☐	Flow cytometry
☒	☐	MRI-based neuroimaging

Antibodies

Antibodies used

PCRP-POU5F1-1D2, MC-813-70 (SSEA-4), Developmental Studies Hybridoma Bank; Goat anti-Mouse IgG (H+L) Cross-Adsorbed Secondary Antibody, Alexa Fluor 488, A11001 Thermo Fisher.

Validation

PCRP-POU5F1-1D2: this antibody was characterized by the Protein Capture Reagents Program, produced by JHU/CDI, https://antibodyregistry.org/search.php?q=AB_2618968.

MC-813-70 (SSEA-4): the use of this antibody on immunofluorescence(IF) has been shown in multiple peer reviewed papers, including The EMBO journal 2.12 (1983): 2355-61., Nature 311.5985 (1984 Oct 4-10): 469-72, Scientific reports 6. (2016 Oct 3): 34476.,Scientific reports 8.1 (2018 Apr 18): 6168. (information provided by the Developmental Studies Hybridoma Bank)

Eukaryotic cell lines

Policy information about cell lines and Sex and Gender in Research

Cell line source(s)

WTC11 (iPSC, Allen institute), WIBR3 (NIH registry #0079)

Authentication

Multiple SNPs matched the published whole-genome sequencing data.

Mycoplasma contamination

The cells were tested for mycoplasma monthly.

Commonly misidentified lines
(See ICLAC register)

No commonly misidentified cell lines were used.
